# Microstratigraphic preservation of ancient faunal and hominin DNA in Pleistocene cave sediments

**DOI:** 10.1073/pnas.2113666118

**Published:** 2021-12-27

**Authors:** Diyendo Massilani, Mike W. Morley, Susan M. Mentzer, Vera Aldeias, Benjamin Vernot, Christopher Miller, Mareike Stahlschmidt, Maxim B. Kozlikin, Michael V. Shunkov, Anatoly P. Derevianko, Nicholas J. Conard, Sarah Wurz, Christopher S. Henshilwood, Javi Vasquez, Elena Essel, Sarah Nagel, Julia Richter, Birgit Nickel, Richard G. Roberts, Svante Pääbo, Viviane Slon, Paul Goldberg, Matthias Meyer

**Affiliations:** ^a^Department of Evolutionary Genetics, Max Planck Institute for Evolutionary Anthropology, Leipzig, D-04103 Leipzig, Germany;; ^b^Archaeology, College of Humanities, Arts and Social Sciences, Flinders University, Adelaide, South Australia 5042, Australia;; ^c^Institute for Archaeological Sciences, Eberhard Karls University of Tübingen, Tübingen, 72074 Tübingen, Germany;; ^d^Senckenberg Centre for Human Evolution and Paleoenvironment, Universität Tübingen, Tübingen, 72070 Tübingen, Germany;; ^e^Interdisciplinary Center for Archaeology and Evolution of Human Behaviour, University of Algarve, 8005-139 Faro, Portugal;; ^f^Sentre For Fremragende Forskning (SFF), Centre for Early Sapiens Behaviour, University of Bergen, Bergen, 5020 Bergen, Norway;; ^g^Department of Human Evolution, Max Planck Institute for Evolutionary Anthropology, Leipzig, D-04103 Leipzig, Germany;; ^h^Institute of Archaeology and Ethnography, Russian Academy of Sciences, Siberian Branch, Novosibirsk 630090, Russia;; ^i^School of Geography, Archaeology and Environmental Studies and Evolutionary Studies Institute, University of the Witwatersrand, Johannesburg, Wits 2050, South Africa;; ^j^Center for Environmental Management of Military Lands, Colorado State University, Fort Collins, CO 80524;; ^k^Centre for Archaeological Science, School of Earth, Atmospheric and Life Sciences, University of Wollongong, Wollongong, New South Wales 2522, Australia;; ^l^Australian Research Council Centre of Excellence for Australian Biodiversity and Heritage, University of Wollongong, Wollongong, New South Wales 2522, Australia;; ^m^Department of Anatomy and Anthropology, Sackler Faculty of Medicine, Tel Aviv University, Tel Aviv 6997801, Israel;; ^n^Department of Human Molecular Genetics and Biochemistry, Sackler Faculty of Medicine, Tel Aviv University, Tel Aviv 6997801, Israel;; ^o^The Dan David Center for Human Evolution and Biohistory Research, Sackler Faculty of Medicine, Tel Aviv University, Tel Aviv 6997801, Israel

**Keywords:** ancient DNA, sediment DNA, sediment curation, soil micromorphology, Denisova Cave

## Abstract

Ancient DNA recovered from Pleistocene sediments represents a rich resource for the study of past hominin and environmental diversity. However, little is known about how DNA is preserved in sediments and the extent to which it may be translocated between archaeological strata. Here, we investigate DNA preservation in 47 blocks of resin-impregnated archaeological sediment collected over the last four decades for micromorphological analyses at 13 prehistoric sites in Europe, Asia, Africa, and North America and show that such blocks can preserve DNA of hominins and other mammals. Extensive microsampling of sediment blocks from Denisova Cave in the Altai Mountains reveals that the taxonomic composition of mammalian DNA differs drastically at the millimeter-scale and that DNA is concentrated in small particles, especially in fragments of bone and feces (coprolites), suggesting that these are substantial sources of DNA in sediments. Three microsamples taken in close proximity in one of the blocks yielded Neanderthal DNA from at least two male individuals closely related to *Denisova 5*, a Neanderthal toe bone previously recovered from the same layer. Our work indicates that DNA can remain stably localized in sediments over time and provides a means of linking genetic information to the archaeological and ecological records on a microstratigraphic scale.

## Significance

DNA preserved in sediments has emerged as an important source of information about past ecosystems, independent of the discovery of skeletal remains. However, little is known about the sources of sediment DNA, the factors affecting its long-term preservation, and the extent to which it may be translocated after deposition. Here, we show that impregnated blocks of intact sediment are excellent archives of DNA. DNA distribution is highly heterogeneous at the microscale in the cave sediment we studied, suggesting that postdepositional movement of DNA is unlikely to be a common phenomenon in cases where the stratigraphy is undisturbed. Combining micromorphological analysis with microstratigraphic retrieval of ancient DNA therefore allows genetic information to be associated with the detailed archaeological and ecological record preserved in sediments.

Sediments are archives of past ecosystems, often preserving remnants of ancient plants and animals. They can also contain evidence of past human activity, such as stone tools, coprolites, and combustion products (1). Sediments are also an important source of ancient DNA (aDNA) (2, 3), allowing the molecular detection of ancient flora and fauna. Automated sample preparation (4) and the application of methods for targeted DNA retrieval (4, 5) have enabled studies of Pleistocene sediment DNA to move beyond the recovery of DNA from species that were well represented in the fossil record of the sites studied, such as mammoth, horse, and bear (6–8), to taxa that were present in much smaller numbers, including hominins (4, 5, 9, 10). This offers exciting new opportunities to investigate the geographical and temporal distribution of Pleistocene hominin groups where few or no skeletal elements are available.

Despite recent technical advances in the isolation and sequencing of aDNA from sediment, the origin of DNA molecules within sediments and the mechanisms enabling their long-time preservation remain poorly understood. While microscopic fragments of bone and teeth are an obvious potential source of aDNA in sediment, DNA can also derive from feces and other organic materials (11, 12). In addition, it has been shown that free extracellular DNA, which may be present in body fluids or released from decaying tissue, can be adsorbed onto mineral particles and organic molecules, such as humic acids, which shield the DNA from nuclease activity (13–15). Current techniques for the isolation of aDNA from sediment are not suited to address these questions because they have been developed for loose (disaggregated) material and lack the spatial integrity needed to study the distribution of aDNA at a microscale and associate it with specific sediment features. To achieve this, we here combine aDNA analysis with microstratigraphic (micromorphological) characterization of undisturbed sediment.

Micromorphological sediment analyses are typically performed on thin sections made from intact blocks of sediment that are removed from stratigraphic sequences (16–18) and then dehydrated and solidified using transparent synthetic resins to preserve the spatial arrangement and integrity of particles (e.g., sand grains) and inclusions (e.g., bone fragments) (19–21). Sediment micromorphology provides information not only on the composition of the sediments but also on their structural integrity and depositional and postdepositional histories. For example, thin-section analysis can reveal whether the freezing and thawing of sediment resulted in the formation of cracks that might have promoted the vertical translocation of particles (22). Additionally, such analyses can reveal the presence of discrete, millimeter-scale layers derived from hominin activities that, in a centimeter-scale sequence, might be separated by tens, hundreds, or thousands of years (23). This information may be crucial for associating DNA sequences with specific parts of a stratigraphic sequence.

Resin impregnation of sediment, however, often involves high-temperature incubation steps both for dehydration of the sample and for resin polymerization (30 to 70 °C for periods of between 8 h and several days) (19–21, 24). This may be detrimental for DNA preservation (25, 26). Furthermore, the polymers used for impregnation may cross-link with DNA molecules, preventing the release of DNA from the sediment, or introduce substances that interfere with DNA extraction or subsequent steps of sample preparation. Although a recent study has shown that lipid biomarkers can be isolated from impregnated blocks (27) and protocols exist for microdrilling of such blocks for geochemical analyses (e.g., stable isotopes) (28), the prospects of DNA retrieval from this type of material have not been investigated.

Here, we assess whether resin impregnation of sediment blocks interferes with the retrieval of aDNA and evaluate ancient mammalian DNA preservation in archived Pleistocene sediment blocks from 13 archaeological sites in Europe, Asia, Africa, and North America. We investigate the micromorphological patterns of aDNA preservation in sediment blocks collected from Denisova Cave in the Altai Mountains (Siberia, Russia), which was occupied by Neanderthals, Denisovans, and modern humans as well as other mammals (10, 29–31) and where aDNA preservation in sediments has been demonstrated (4, 5, 10). We show that impregnated sediment allows the retrieval and contextualization of aDNA from a variety of organisms, including archaic hominins.

## Results

### Effect of Resin Impregnation on aDNA Recovery.

To test whether resin impregnation of sediment interferes with DNA preservation and recovery, we produced “miniblocks” from seven Pleistocene sediment samples with known preservation of ancient mammalian DNA (4, 5) [one sample each from Chagyrskaya Cave, Denisova Cave, Trou Al’Wesse, Vindija Cave, and El Sidrón and two from Galería de las Estatuas (see *Materials and Methods*)]. Between 5 and 17 g of loose sediment were dehydrated at 40 °C for 4 h, and three subsamples of ∼0.5 g from each were turned into miniblocks by impregnation using polyester resin (*SI Appendix*, Figs. S1 and S2 and Dataset S1). The miniblocks were hardened at 60 °C for 24 h and then powdered using a dentistry drill. DNA was extracted and converted to DNA libraries (32) using ∼50 mg of powder from each miniblock as well as ∼50 mg of the loose and dehydrated sediment of each sample.

**Fig. 1.**
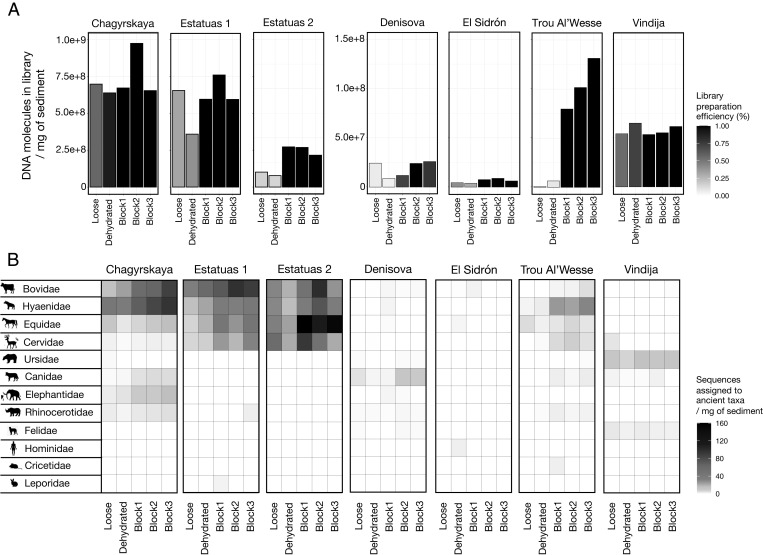
DNA recovery from loose, dehydrated, and freshly impregnated (“miniblock”) subsamples of seven sediment samples from six archaeological sites. (*A*) Number of molecules recovered in the DNA libraries per milligram of sediment. The shade of the bars indicates library preparation efficiency. (*B*) Assignments of ancient mtDNA fragments to mammalian families and number of sequences obtained from each family per milligram of sediment.

As determined by qPCR (32), the number of library molecules obtained per milligram of sediment ranged from 9.7 × 10^5^ to 9.9 × 10^8^ across all samples and treatments (Fig. 1*A* and Dataset S1). Unexpectedly, in all but one sample (from Vindija Cave), the impregnated material yielded more DNA than the unimpregnated material. In the most extreme case (Trou Al’Wesse), the number of molecules retrieved from the impregnated material was, on average, 107 times higher than from the loose material and 16 times higher than from the dehydrated material. Additionally, library preparation efficiency [estimated from the conversion rate of a synthetic oligonucleotide added to the DNA sample before library preparation (32, 33)] was lower for the loose and dehydrated material than for the impregnated material for most of the samples (Fig. 1*A* and Dataset S1). The impregnated subsamples thus contained lower concentrations of substances that inhibit library preparation, such as humic acids, which often occur in sediments, presumably because the sampled material consisted only partly of sediment (and partly of resin).

We next compared the mammalian taxonomic composition in the loose, dehydrated, and impregnated subsamples by hybridization capture with a probe set containing 242 mammalian mitochondrial DNA (mtDNA) genomes (34). MtDNA sequences from the enriched libraries were assigned to mammalian families and evaluated for the presence of deamination-induced cytosine-to-thymine (C-to-T) substitutions at their ends (4), which are expected to be present in genuine aDNA sequences (35, 36). Ancient mammalian DNA was recovered from all subsamples, except for some of the loose and impregnated subsamples from El Sidrón (Spain), for which the yield of DNA was close to the detection limit (Fig. 1*B* and Dataset S1). At the other sites, there was little variation in the taxonomic composition, with the most dominant families being recovered from all subsamples. No aDNA was detected in sediment-free resin samples taken in proximity to the impregnated sediment in the miniblocks (Dataset S1). Thus, under the experimental conditions used, resin impregnation had no negative effect on the preservation of aDNA or our ability to extract it.

### DNA Preservation in Archived Blocks.

Extensive archives of resin-impregnated micromorphology blocks have been collected and stored in recent decades and could now potentially be used for genetic analyses. It is unclear, however, whether long-term storage of blocks at room temperature interferes with DNA preservation. In addition, blocks were prepared in different laboratories using different resins and protocols for dehydration and polymerization, detailed records of which are often not available. To test the suitability of archived blocks for DNA analysis, we analyzed 294 samples drilled from 47 blocks collected over the past 40 y from 13 archaeological sites across four continents, representing sediments deposited from the Middle Pleistocene to the early Holocene (*SI Appendix*, Supplementary Text, Fig. S3, and Table S1).

We recovered ancient mammalian mtDNA from 23 of the 47 blocks tested (*SI Appendix*, Fig. S3 and Datasets S2–S4), including all six blocks from Denisova Cave (Russia), all three blocks from Hohle Fels (Germany), 12 of 14 blocks from La Ferrassie (France), one of two blocks from Geißenklösterle (Germany), and a block of Holocene sediment from Aşıklı Höyük (Turkey). Neither of the blocks analyzed from the Pleistocene sites of Pech-de-l’Azé IV (France) or Schöningen (Germany) yielded ancient mammalian DNA, nor did the three Pleistocene blocks from Kebara (Israel), the block from Sierra Diablo (United States), or any of the 15 blocks collected from sites in Africa (Bizmoune in Morocco; Blombos Cave, Klasies River Mouth, and Klipdrift Shelter in South Africa). These results align well with the known temporal and geographical limits of aDNA recovery from skeletal material, suggesting that preservation conditions at the sites, rather than the process of resin impregnation, are the primary factor limiting the retrieval of aDNA from the blocks studied.

### Spatial Distribution of Ancient Mammalian DNA in Sediment.

**Fig. 2.**
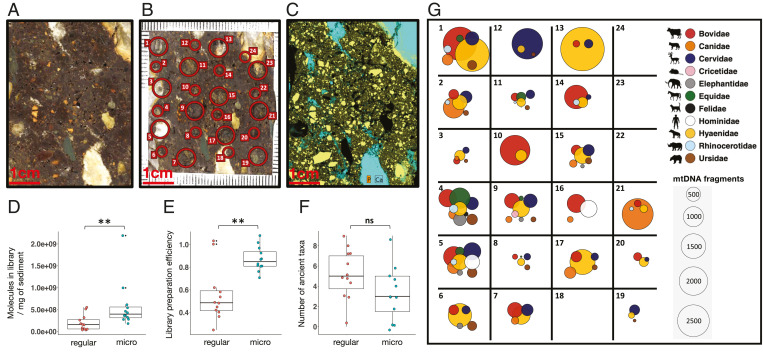
Microstratigraphic distribution of ancient mammalian DNA in impregnated sediment. (*A*) Macro scan of the selected surface on block DCE5 from Denisova Cave. (*B*) Locations of the regular and micro sampling spots from which DNA was extracted (size of sample indicated by circle size). (*C*) A µXRF surface scan for P (orange) and Ca (aqua) produces a distribution map of calcium phosphate (yellow) that indicates fragments of hydroxyapatite from bone, coprolite, and phosphatized limestone. (*D*–*F*) Boxplots comparing the number of library molecules recovered from regular samples and microsamples, the efficiency of library preparation, and the number of ancient mammalian taxa identified. Boxes indicate the mean and interquartile range, whiskers show the minimum and maximum values, and outliers are marked with black dots. Differences were tested for significance using an unpaired two-sample Wilcoxon test (*D* and *E*) and an unpaired two-sample *t* test (*F*) and considered significant if the *P* value was smaller than the significance level alpha = 0.05 (**P < 0.01 < ns [not significant]). (*G*) Assignments of mtDNA sequences to ancient mammalian taxa (regular samples and microsamples are indicated by odd and even numbers, respectively).

To determine the preservation and composition of DNA in sediments at a microstratigraphic scale, we drilled into a resin-impregnated block straddling the interface of layers 11.3 and 11.4 in the East Chamber of Denisova Cave [block DCE5 (37); Fig. 2*A* and *SI Appendix*, Fig. S4] and removed 12 samples ranging in weight from 20.4 to 41.6 mg (30.1 mg, on average) (Fig. 2*B*, *SI Appendix*, Fig. S5, and Dataset S3); we henceforth refer to these as “regular” samples. In addition, we explored the possibility of retrieving aDNA from smaller samples (subsequently referred to as “microsamples”), obtained by drilling 12 holes with diameters of ∼1 mm, which yielded between 1.5 and 8.1 mg of powdered material (3.4 mg on average) (Fig. 2*B*, *SI Appendix*, Fig. S5, and Dataset S3). The “regular” samples and the microsamples were collected alternately in a grid-like pattern (Fig. 2*B* and *SI Appendix*, Fig. S5). Prior to sampling, the distribution of small particles (e.g., organic inclusions) visible on the cut face of the block was recorded using a flatbed scanner (38) (Fig. 2*A* and *SI Appendix*, Fig. S5). In addition, nondestructive elemental analysis was performed using micro X-ray fluorescence (µXRF) (28, 39) (Fig. 2*C*), which revealed the near-ubiquitous presence of small particles that contain both calcium (Ca) and phosphorus (P). These likely represent small fragments of bone and/or coprolite and possibly pieces resulting from weathering of limestone clasts.

The number of DNA molecules incorporated in the libraries was, on average, 2.9-fold lower per mg of sediment for the regular samples compared to the microsamples (Fig. 2*D* and Dataset S3). Library preparation efficiencies were also lower for the regular samples, indicating that the coextraction of inhibitory substances reduced DNA yields for the latter (Fig. 2*E*). Notably, ancient mammalian DNA was successfully recovered from 9 of 12 microsamples (compared to 11 of 12 regular samples), demonstrating that DNA analysis of impregnated sediments is possible even from extremely small (∼1 to 8 mg) amounts of material.

There were no significant differences in the number of ancient mammalian taxa recovered from regular samples and microsamples (Fig. 2*F*), with both containing DNA from up to nine mammalian families (Fig. 2*G* and Dataset S3). However, the taxa and their relative abundance differ greatly among neighboring samples, with the most abundant family in a given sample rarely recapitulating that of its neighbors (Fig. 2*G* and Dataset S3). To characterize the spatial dispersion of each mammalian family, we used the distance between samples that yielded DNA from the respective taxon to compute the Nearest Neighbor value (*Rn*). This measure produces values between 0 and 2.15, indicating whether the DNA of a taxon cluster in the block (*Rn* ∼0) is distributed randomly (*Rn* ∼1) or uniformly (*Rn* ∼2.15) (40, 41). *Rn* values for the eight taxa identified in three or more samples range between 0.39 and 0.69, suggesting a random (i.e., nonuniform) distribution with a tendency toward clustering (*SI Appendix*, Table S2). Thus, the taxonomic composition of ancient mammalian DNA in sediment is highly heterogeneous on a microscale in the block studied here.

### Source of Ancient Mammalian DNA in Sediment.

The recovery of aDNA from microsamples of impregnated sediment opens up the possibility to investigate DNA preservation in specific, morphologically identifiable sediment particles or clasts, which we term “microfeatures.” Using surface scans of five blocks from Denisova Cave [DCM1B, DCM2A, DCM2B, and DCM2C from layers 11.2, 12.2, 12.3, and 14.1, respectively, in the Main Chamber and DCE5C (a part of DCE5 previously removed for the preparation of thin sections) from the interface of layers 11.3 and 11.4 in the East Chamber (37); *SI Appendix*, Fig. S4], we identified microfeatures sufficiently large (greater than ∼0.5 mm) for targeted sampling (*SI Appendix*, Figs. S6–S10). These included features identified by optical microscopy as coprolites (*n* = 109), bones (*n* = 10), unidentifiable grains that are likely bones or coprolites (*n* = 21, omitted in subsequent analysis), and inorganic components (limestone, chert, schist, or clay aggregate grains; *n* = 11). In addition, we collected sediment “matrix samples” at locations where no microfeatures were discernible on the cut face (*n* = 37) as well as by untargeted sampling of morphologically uncharacterized sediment up to ∼1 cm below the surface of the block (*n* = 8), yielding a total of 196 samples weighing between 0.7 and 49.1 mg (4.2 mg, on average) (*SI Appendix*, Fig. S11 and Dataset S4).

The average number of DNA molecules recovered from the different samples was similar across all blocks, with the exception of DCM2B, in which yields were lower (*SI Appendix*, Fig. S12*A*) despite high library preparation efficiencies (*SI Appendix*, Fig. S12*B*). The poor DNA preservation in DCM2B may be linked to diagenetic transformations (evidenced by the presence of phosphatic rinds around limestone clasts and dissolution of calcium carbonate), which were previously identified in thin sections from this block but not in the other blocks (37).

**Fig. 3.**
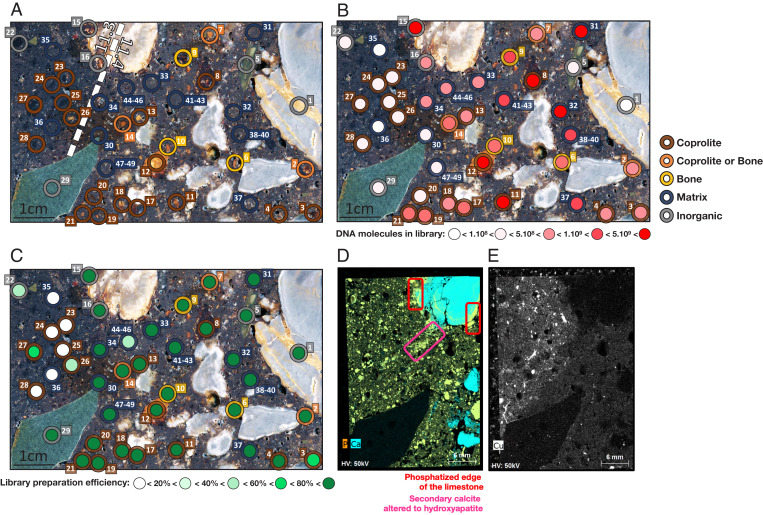
Targeted sampling of microfeatures from block DCE5C. (*A*) Surface scan with sampling locations and layer designations. (*B*) Number of library DNA molecules recovered from each sample. (*C*) Library preparation efficiencies. (*D*) µXRF surface scan for P (orange) and Ca (aqua) produces a distribution map of calcium phosphate (yellow) that indicates fragments of hydroxyapatite from bone and coprolite as well as phosphatized limestone (red frames) and secondary calcite (magenta frame). (*E*) µXRF surface scan for Cu (white).

Block DCE5C crosses the interface between two layers (Fig. 3*A* and *SI Appendix*, Fig. S4) and exhibits a pattern of DNA preservation that appears to be layer specific. Samples from layer 11.3 yielded fewer molecules than samples from layer 11.4 (Fig. 3*B*) and show evidence for the presence of inhibitory substances (Fig. 3*C*). The elemental composition map of the cut face of this block revealed no differences in the prevalence of Ca- and P-containing microfeatures between these layers (Fig. 3*D*; see *SI Appendix*, Fig. S13 for the complete elemental analysis). However, we observed evidence of secondary phosphatization in layer 11.3, suggesting that some of the microfeatures sampled could be phosphatized limestone and secondary calcite altered to hydroxyapatite rather than bone or coprolite. Furthermore, we detected a higher concentration of copper (Cu) in layer 11.3 than in layer 11.4 (Fig. 3*E*). Cu in soil is mainly bound to humic acid, which is known to inhibit enzymatic reactions (42–44). Both secondary phosphatization and enrichment in Cu have also been linked to the presence of bat guano (45, 46), the chemical composition of which is likely detrimental for long-term DNA preservation.

**Fig. 4.**
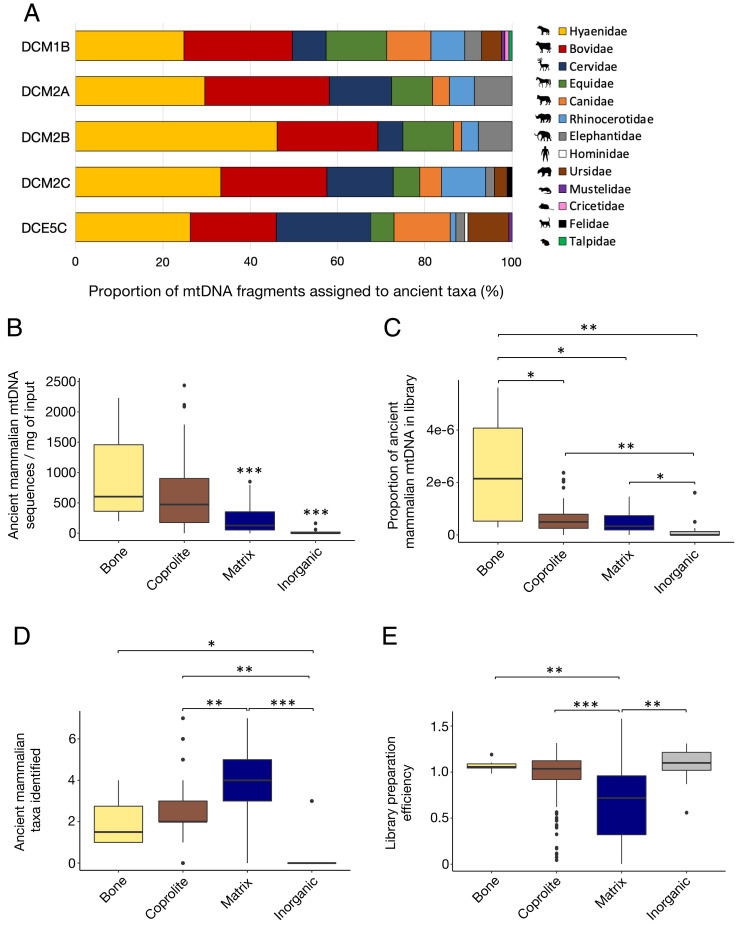
Taxonomic composition and yield of ancient mammalian DNA from sediment matrix samples and microfeatures from five micromorphology blocks from Denisova Cave. (*A*) Taxonomic composition of ancient mammalian mtDNA fragments recovered from each block. (*B*–*E*) Boxplots comparing the number of ancient mammalian mtDNA fragments recovered per milligram of sample from the sediment matrix and each type of microfeature, the proportion of library molecules originating from ancient mammalian mtDNA fragments, the number of ancient mammalian taxa identified, and library preparation efficiencies. Boxes indicate the mean and interquartile range, whiskers show the minimum and maximum values, and outliers are marked with black dots. Differences were tested for significance using an unpaired two-sample Wilcoxon test and considered significant if the *P* value was smaller than the significance level alpha = 0.05 after correction was applied from multiple comparisons using the Bonferroni method (***P < 0.0001 < **P < 0.001 < *P < 0.008).

Ancient mammalian mtDNA was recovered from 86, 94, 47, 89, and 70% of the samples from blocks DCM1B, DCM2A, DCM2B, DCM2C, and DCE5C, respectively (*SI Appendix*, Fig. S14). This DNA is derived from a total of 13 mammalian families (Fig. 4*A*), of which up to 7 were present in individual samples (Dataset S4). All identified families are known from the zooarchaeological records of Denisova Cave (30, 37, 47, 48).

Inorganic features, sampled mostly by drilling the inner part of rock grains (ranging from a few millimeters to a few centimeters in diameter), showed the lowest rate of aDNA recovery as expected [with 2 positive samples out of 11 (Dataset S4)] but not a rate of zero, possibly because sampling could not always be accurately confined to the targeted microfeatures and may have included surrounding sediment matrix in some cases. In contrast, aDNA was recovered from all 10 microsamples that targeted bone fragments, which also yielded the highest number and concentration of mammalian mtDNA molecules among the microfeatures analyzed (Fig. 4 *B* and *C*). Bone microsamples also contained DNA from a smaller number of taxa than coprolite or sediment matrix samples (Fig. 4*D* and *SI Appendix*, Fig S15). Specifically, five bone samples yielded DNA from a single family, two from two families, and three from three or four families (*SI Appendix*, Fig. S16 and Dataset S4). When considering only the dominant families (i.e., families representing at least 10% of the identified sequences), only one or two families are identified in the bone samples. When two families are present, these always include Hyaenidae and a herbivore (*SI Appendix*, Fig. S16). Thus, these results are compatible with the idea that bone fragments contain DNA from a single taxon, except when they were ingested by hyenas and adsorbed DNA from their digestive tracts in the process.

Significantly more mammalian aDNA was recovered from the coprolite microsamples than from the sediment matrix samples (Fig. 4*B*), demonstrating that coprolites represent a rich source of aDNA in sediment. Coprolite samples also contain DNA from significantly fewer taxa than sediment matrix samples (Fig. 4*D*). Analyses of the cut face of the blocks by µXRF revealed the presence of Ca- and P-rich finely divided bone and/or coprolite particles forming a part of the sediment matrix of the blocks from Denisova Cave (*SI Appendix*, Fig. S13). We cannot determine, therefore, whether adsorption of mammalian aDNA to mineral particles contributes to the aDNA recovered from the matrix samples or whether mammalian aDNA in the matrix originated exclusively from bones and/or coprolites present as both fragments and in finely comminuted form. It also remains unclear whether bone and coprolite microfeatures contain more DNA than the sediment matrix because DNA retrieval from the latter may have been impaired by the coextraction of inhibitory substances (Fig. 4*E*).

### Hominin aDNA in Impregnated Sediment.

Of the 220 sediment block samples analyzed from Denisova Cave, a regular sample and a microsample from block DCE5 and one sediment matrix sample obtained by untargeted drilling from block DCE5C yielded hominin DNA after enrichment for mammalian mtDNA (Fig. 2*G* [subsamples 5 and 16] and Fig. 3*A* [subsample 46]). Small traces of hominin mtDNA may not always be detected with this probe set (4), so we further enriched all libraries specifically for hominin mtDNA. This did not lead to the identification of hominin aDNA in additional samples but yielded more hominin mtDNA fragments from the three DCE5 samples.

To maximize the number of fragments available for analysis, we prepared additional libraries from the remaining aliquots of DNA extracted from these samples and extracted DNA from the undigested material remaining after the first extraction. This resulted in a total of 3,005, 5,991, and 7,044 sequences per sample (*SI Appendix*, Table S3), of which 72.8, 53.4, and 73.2%, respectively, could be assigned to Neanderthals and the remainder to present-day human contamination (*SI Appendix*, Table S4). After filtering for fragments with evidence of deamination-induced C-to-T substitutions at their three first or last bases (see *SI Appendix*, Figs. S17–S19 for substitution profiles), we retained 1,009, 1,086, and 2,271 fragments, respectively (between 70 and 1,156 per library, median of 474) (*SI Appendix*, Table S4). These values are higher than those obtained for most of the samples containing hominin DNA in a previous study of the Denisova Cave sediments (10) (median of 62 deaminated fragments per library).

**Fig. 5.**
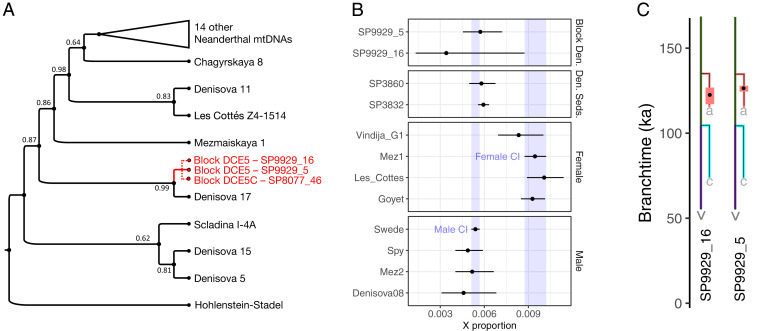
Neanderthal DNA in block DCE5. (*A*) Neighbor-joining phylogenetic tree of 23 previously published Neanderthal mtDNA genomes and the most complete mtDNA consensus sequence of the regular sample SP9929_5 from block DCE5. Bootstrap supports (500 replicate trees) are shown next to the branches. The tree is rooted using the divergent mtDNA of the Hohlenstein-Stadel Neanderthal. The branches leading to the mtDNA consensus sequences of the other two samples from block DCE5 (microsample SP9929_16 and sediment matrix sample SP8077_46 in dashed lines) have been superimposed based on phylogenetic trees relating each of them to the same set of 23 Neanderthal mtDNA genomes (*SI Appendix*, Fig. S23–S25). (*B*) Sex determination for samples SP9929_5 (regular sample) and SP9929_16 (microsample) based on the coverage of the X chromosome and the autosomes. In the second to fourth subpanels, the same approach was used with previously published data as controls from two sediment samples from Denisova Cave (5) from female and male ancient hominin skeletal samples (50, 72, 73) and from a modern male individual (“Swede”) (71). Male and female CI bands (purple) denote male and female skeletal samples with the narrowest CI. (*C*) Population split time estimates for the same two samples from block DCE5. The black dots indicate the maximum likelihood estimates of divergence dates from a Neanderthal population tree inferred using the high-coverage genomes of the Vindija 33.19 individual (v; purple line), the Chagyrskaya 8 individual (c; blue line) and the Altai Neanderthal “Denisova 5” individual (a; red line) with 95% block bootstrap CIs indicated by the thick lines.

Reconstruction of mtDNA genomes using only deaminated fragments resulted in an internally consistent consensus sequence for each sample (*SI Appendix*, Figs. S20–S22) covering 54, 79, and 76% of the complete mtDNA reference genome (*SI Appendix*, Table S5). The three sequences cluster with the mtDNA of the Neanderthal *Denisova 17*, a bone fragment from the East Chamber, layer 12 (49), which underlies the layer from which DCE5 was collected (Fig. 5*A* and *SI Appendix*, Figs. S23–S25 and Table S6). The three sequences show only one divergent position in which deaminated fragments from the regular samples and microsamples (represented by two and three sequences, respectively) carry an adenine base, whereas all five fragments from the sediment matrix sample carry a guanine base (*SI Appendix*, Fig. S26). Thus, while the mtDNA in each sample may originate from a single individual, at least two Neanderthal individuals contributed DNA to the samples from block DCE5.

We next enriched the libraries with a probe set targeting 1.6 million single nucleotide polymorphisms (SNPs) in the hominin nuclear genome (5) and obtained sufficient data from only the regular sample and the microsample for further analysis (*SI Appendix*, Table S7). The sequence coverage of X chromosomal and autosomal SNPs suggests that the aDNA in both samples derived from male individuals (Fig. 5*B*). A maximum likelihood method (5) indicates that both samples are more closely related to the Neanderthal *Denisova 5* (the “*Altai Neanderthal*”) (50) recovered from the same layer (11.4) as DCE5 than to other Neanderthals (Fig. 5*C*) and estimates population split times that slightly predate or are concurrent with the optical dates of the layer [105,000 to 120,000 thousand years ago (30)]. Thus, the mtDNA, sex, and lineage assignment are compatible with (but provide no conclusive evidence for) the DNA in these two samples originating from a single male individual.

## Discussion

The importance of archiving sediment samples for future genetic and other analyses is becoming increasingly recognized, particularly for sites that have been extensively excavated. In the past, excavated sediments were often not retained for analysis, but notable exceptions are resin-impregnated blocks of undisturbed sediment collected from stratigraphic sections for the specific purpose of understanding the formation of sites and their occupational history. By preserving the original physical arrangement of individual components (e.g., sediment matrix and inclusions), impregnated sediments retain the spatial architecture and contextual relationships of features of interest as well as any postdepositional modifications. Here, we show that such intact blocks are excellent sources of aDNA despite years or decades of storage at ambient temperature and represent a previously unused repository of genetic information.

Our study directly links sediment aDNA to the spatially resolved archaeological microcontext and therefore provides a means to address a critical problem associated with the analysis of aDNA from sediment: the possibility of translocation of DNA in the stratigraphy. This may occur either through the movement of DNA-containing particles or by movement of DNA in solution (3, 51–54). Extraction of DNA from impregnated sediments allows these possibilities to be evaluated and mitigated. First, micromorphological analyses of thin sections of sediment blocks allow postdepositional movement of particles to be visually identified (52, 55). Subsequent sampling of the blocks for genetic analyses can then target areas that show no or few signs of disturbance and diagenesis or syndepositional hominin activities such as burning. Second, translocation of DNA could occur, for example, as a result of percolating water or of repeated deposition of urine (3, 54, 56), which should presumably result in a more homogeneous distribution of the DNA of the taxa of interest in the stratigraphy. Thus, when the DNA from adjacent samples shows heterogeneity in taxonomic composition, as observed in Denisova Cave, movement of DNA through leaching is unlikely to have occurred to a large extent.

Our approach shows that bone fragments and coprolites are rich sources of mammalian aDNA in Paleolithic deposits. Given the presence of finely comminuted bone and coprolite microparticles in the sediment matrix of the blocks analyzed here, it is likely that the matrix samples did not consist solely of inorganic material. It is not possible, therefore, to ascertain whether adsorption of DNA onto mineral grains contributes to its long-term preservation in the sediment. Moreover, the substantial heterogeneity observed in the taxonomic composition of mammalian DNA at sampling locations only a few millimeters apart suggests that the DNA is primarily concentrated in organic inclusions (e.g., bone and coprolite) rather than being uniformly distributed throughout the sediment matrix. The adsorption of free extracellular DNA from feces, bodily fluids, or decomposing cellular tissue onto mineral grains does not, therefore, appear to play a major role in the accumulation of mammalian aDNA in the sediments at Denisova Cave.

In a recent study, ∼24% of more than 700 loose sediment samples from Denisova Cave yielded hominin aDNA (10), whereas the success rate for the impregnated blocks examined here is an order of magnitude lower (3 of 220 samples). This difference is compatible with the highly localized occurrence of hominin DNA in undisturbed sediments. However, the extraction of aDNA from hominins or other rare taxa from disaggregated sediment samples (4, 5, 9, 10) can be fruitfully complemented by targeting “hot spots” of aDNA preserved in sediment blocks as demonstrated here.

From a practical perspective, we recommend a two-step approach to maximize the yield of aDNA information from sediments: 1) an initial screening of loose sediment samples from throughout a stratigraphic section to obtain broad spatial and temporal coverage of aDNA survival for the taxa of interest and to identify those parts of the stratigraphy of greatest potential for further sampling and 2) a targeted extraction of aDNA from identified microfeatures in impregnated blocks to increase the chance of recovering a high-yield sample of DNA. Our data suggest that hominin DNA in impregnated sediments is commonly localized rather than being distributed uniformly throughout the sediment matrix and that a localized sample is more likely to yield DNA originating from a single individual than is the case for loose sediment samples. Sampling of micromorphology blocks, therefore, offers the prospect of increasing the quantity of aDNA that can be recovered from sediments, especially for hominins and other rare taxa. Consequently, we urge the systematic preparation of resin-embedded sediment blocks at all excavations.

## Materials and Methods

### Preparation and Sampling of Miniblocks.

In a clean-room facility dedicated to the analysis of aDNA at the Max Planck Institute for Evolutionary Anthropology in Leipzig, samples of between 5 and 17 g of loose sediment from six Paleolithic sites [selected based on their known preservation of ancient mammalian DNA (4, 5); *SI Appendix*, Supplementary Text and Dataset S1] were transferred to plastic weigh boats and dehydrated for 4 h at 40 °C.

For impregnation, subsamples of ∼500 mg of dehydrated sediment were transferred to 2 mL Eppendorf tubes containing a bed of ∼1.5 mL of Viscovoss N 55S unpromoted polyester resin diluted in acetone (ratio of 7:1) hardened by polymerization catalyzed with methyl ethyl ketone peroxide (MEKP) (0.05 mL for 100 mL of resin/acetone mixture) and incubation at 60 °C for 24 h. The ∼500 mg dehydrated sediment subsamples were impregnated at room temperature by repeatedly adding volumes of resin/acetone/MEKP (resin mix) that were large enough to cover the sediment. The impregnation time varied from sample to sample from several hours to a few days. Once the resin stopped penetrating the sample, the tubes were filled with an additional volume of resin mix up to the formation of a convex meniscus at the top. The impregnation was considered complete when the convex meniscus on top of the tube remained stable for 24 h. The tubes were then incubated at 60 °C for 24 h. Hardening of the sediment miniblock samples (*SI Appendix*, Fig. S2) was completed by incubation for 4 d at room temperature.

The excess of hardened resin on top of the miniblock was sampled using a sterile dentistry drill prior to the sampling of the impregnated sediment and used as resin control samples without sediment. For each miniblock, all of the impregnated sediment was drilled and weighed. The sediment/resin mass ratio was determined for each impregnated sample by dividing the weight of the dehydrated sample before impregnation by the weight of the drilled sample after impregnation. This ratio was used to estimate the proportion of sediment in the ∼50 mg of drilled impregnated sample used for DNA extraction (Dataset S1). Negative controls for the miniblocks were prepared using a resin-only mix and sampled alongside the sediment miniblocks.

### μXRF of Block Surfaces.

Elemental distribution maps of the cut faces of micromorphology blocks were produced using a Bruker M4 Tornado μXRF analyzer equipped with a 50 kV rhodium X-ray tube and dual silicon drift detectors. Analyses were conducted under full vacuum, without filters, and with an anode current of 600 µA. The spot size was ∼20 µm, and maps were produced with pixel spacing of 30 to 100 μm depending on the desired resolution. Dwell times ranged from 5 ms per pixel for overview scans to 2,000 ms per pixel for maps of trace elements. Maps were produced for Al, Si, P, S, K, Ca, Ti, Mn, Fe, Cu, Zn, Sr, Zr, Rb, Mg, Cr, and Pb; elements deemed significant were based on peaks produced within the area(s) of interest. Peak deconvolution was applied prior to generating distribution maps, including the overlay maps of P and Ca used to map the distribution of limestone, secondary carbonate, and calcium phosphates (Figs. 2 and 3 and *SI Appendix*, Fig. S13). We did not observe striking differences in DNA content between samples from the same micro/macro strata removed before μXRF analysis (Layer 11.4 block DCE5C, *SI Appendix*, Fig. S10 and Dataset S4) and after (Layer 11.4 block DCE5, *SI Appendix*, Fig. S5 and Dataset S3), which is consistent with the idea that the radiation produced with these instrument settings is well within the limits considered safe for DNA preservation (57).

### Annotation of Microfeatures on Cut Faces of Impregnated Blocks from Denisova Cave.

The faces of selected blocks were scanned using an Epson V600 flatbed scanner at high resolution (1,200 dots per inch [DPI]). These scans were used to visually identify areas and points of interest for subsequent microdrilling and aDNA analysis. Between 28 and 31 points were chosen from each face with a focus of targeting 1) bone, 2) coprolite or organic phosphate, and 3) rock fragment. In addition, sediment matrix was sampled in areas where no microfeatures were visible.

### Sampling of Sediment Blocks Prepared prior to This Study.

Prior to sampling, the selected surface area of each block was cleaned with tissue paper soaked in 0.5% bleach to remove present-day human DNA contamination. Residual traces of bleach were removed by wiping the surface three times with tissue papers soaked in high performance liquid chromatography (HPLC)-grade water. Sediment powder was removed by drilling into the block using a sterile dentistry drill with a tungsten carbide drill bit. Between samplings, the surface was wiped with 70% ethanol to remove any traces of residual powder.

### DNA Extraction and Library Preparation.

For the miniblock experiment, DNA was extracted from ∼50 mg of loose, dehydrated, and impregnated sediment and from resin-only controls using a manual silica column-based protocol optimized for the recovery of aDNA molecules (58, 59) in the implementation with buffer “D” as described elsewhere (60). A total of 10 µL of each extract (from a total volume of 50 µL) was converted to double-indexed single-stranded DNA (ssDNA) libraries using the automated ssDNA 2.0 protocol (32). This protocol includes the spike-in of a synthetic oligonucleotide into each reaction (33) to gauge the efficiency of library preparation.

All other DNA extractions were performed using a bead-based automated version of the same DNA extraction method (60). For the regular samples and microsamples from the blocks of Denisova Cave, the volume of lysis buffer was reduced to 300 µL (default is 1 mL). Irrespective of the volume of lysis buffer used, DNA was purified from 150 µL lysate and the complete volume of DNA extract used as input for single-stranded library preparation. Extraction and library negative controls were carried through all steps of the experiment. Sample quantities and the number of library molecules recovered from the samples, controls, and spike-in are provided in Datasets S1–S4.

### Hybridization Capture.

The libraries were enriched either for mammalian mtDNA using a probe set of 242 taxa (34), for human mtDNA using a probe set covering the entire human mtDNA genome (61, 62), or for human nuclear DNA using a probe set targeting 1.6 million informative SNPs (5). For mtDNA capture, libraries were individually enriched in two consecutive rounds of on-bead hybridization capture (4, 61) before being pooled and sequenced on a MiSeq (Illumina) in 2 × 76-cycle paired-end runs with two index reads. For the nuclear SNP capture, libraries were enriched by two consecutive rounds of in-solution hybridization capture (62) before being pooled and sequenced on a HiSeq2500 (Illumina) in 2 × 76-cycles paired-end configuration with two index reads.

### Data Processing and Taxonomic Sequence Identification.

After sequencing, base calling was performed using Bustard (Illumina). Sequences that did not exactly match the expected index combinations were discarded. Adapter sequences were trimmed and overlapping paired-end reads were merged using leeHom (63). Assignments of sequences from the mtDNA enriched libraries to mammalian families were made using a pipeline developed previously (4), which is based on basic local alignment search tool (BLAST) (64) and MEtaGenome ANalyzer (MEGAN) (65). The presence of aDNA was inferred separately for each taxon based on the frequency of terminal C-to-T substitutions in sequence alignments (4). Lineage assignments of the hominin DNA in the samples were made based on the sharing of the ancestral or derived state at diagnostic positions in the mtDNA genome that differentiate the mtDNAs of modern humans, Neanderthals, Denisovans, and Sima de los Huesos hominins (66), as described previously (10).

Overlap-merged sequences from the libraries enriched for hominin nuclear DNA were processed as in Vernot et al. (5). Briefly, reads were mapped to the human reference genome hg19 available from the University of California, Santa Cruz, genome browser using Burrows-Wheeler Aligner (67) with parameter « −n 0.01 −o 2 –l 16500 » (68). PCR duplicates were collapsed into single sequences by consensus calling using bam-rmdup (https://github.com/mpieva/biohazard-tools). Sequences shorter than 35 bases or with a mapping quality lower than 25 were discarded. We then restricted the analyses to reads overlapping targeted sites. Remaining sequences were assigned to the National Center for Biotechnology Information (NCBI) Taxonomy using Kraken (69). For both all reads and reads assigned to the Primate clade, we calculated the proportion of nonhominin faunal mis-mapping using hominin diagnostic alleles. For all libraries, both the all reads and Primate groups had point estimates of faunal mis-mapping less than 2% (*SI Appendix*, Table S7). We therefore used the less restrictive all reads set of sequences for all analyses. We next looked for evidence of aDNA damage as measured by C-to-T substitutions to the reference genome at the three first or last bases (deaminated sequences). Five libraries had significant signatures of aDNA damage (C-T mismatch proportion > 10% on both 5′ and 3′ ends, 95% binomial CI). These libraries originated from the regular sample and the microsample from block DCE5. For all subsequent analyses, the libraries of the same sample were merged (*SI Appendix*, Table S7).

### Nearest Neighbor Value.

For each sample positive for a specific taxon, we measured the distance to the nearest sample positive for the same taxon over the 14 cm^2^ sampled surface area of block DCE5 (*SI Appendix*, Table S2). We inferred the clustered, random, or uniform (homogeneous) distribution of the ancient mammalian taxa identified in at least three samples by measuring the Nearest Neighbor value (*Rn*) for each taxon using the following equation:

$$Rn=\;\frac{D\;(\mathrm{Obs})}{0.5\;x\sqrt{\frac{a}{n}}}$$

where *Rn* is Nearest Neighbor value, D(Obs) is the mean value of the nearest neighbor distance, a is the surface area sampled, and *n* is the number of positive samples

### Human mtDNA and Nuclear Analysis.

Unique hominin mtDNA fragments from libraries prepared from the same DNA extracts were merged. Because of the presence of present-day human DNA contamination in the samples, we restricted the analysis to sequences showing evidence of C-to-T substitutions to the reference genome at the three first or last bases (deaminated sequences). We determined consensus bases by majority call at positions covered by at least two sequences if at least 66% of the fragments carried the same base. The consensus sequences were aligned to a dataset of 23 previously published Neanderthal mtDNA genomes described in Brown et al. (49). Pairwise differences between mitochondrial genome sequences and neighbor joining trees were inferred using MEGA X (70).

Internal consistency of the mtDNA sequences from each sample was evaluated by determining the support of the consensus base at positions covered by at least 10 deaminated DNA fragments. Positions with a consensus base support lower than 80% were taken as indication for the presence of more than one mtDNA and visually inspected using Geneious Prime 2021.0.3 (https://www.geneious.com). Sequence alignments at all these positions were compatible with an accumulation of deamination-induced substitutions close to the ends of DNA fragments.

For nuclear data analyses, we merged data of the libraries positive for ancient hominin nuclear DNA from the same sample (A27993, A30911, A30913 and A28004, A30912, respectively) (*SI Appendix*, Table S7). We first considered the proportion of deaminated reads that originate from the X chromosome versus autosomal chromosomes in order to determine the sex of the individual(s) who contributed DNA to the samples as well as skeletal control samples [Swede (71), Vindija G1, Mezmaiskaya 2, Spy 94a, Goyet Q56-1, Les Cottés Z4-1514 (72), Mezmaiskaya 1 (50), and Denisova 8 (73)] and sediment samples SP3860 and SP3832 from Denisova Cave (5). In both cases, these proportions are consistent with the DNA originating primarily from one or more male individuals. We next tried to place each sample on the Neanderthal phylogeny, as defined by the three high-coverage Neanderthal genomes. We used a maximum likelihood method to estimate the branch and split time from that branch for each sample (5). For both samples, the maximum likelihood split time, and the 95% block-bootstrap CI for that estimate, fell on the Altai Neanderthal branch.

## Supplementary Material

Supplementary File

Supplementary File

Supplementary File

Supplementary File

Supplementary File

## Data Availability

Aligned DNA sequence data of the captured libraries have been deposited in the European Nucleotide Archive under accession PRJEB46683.
