# Supplementary material for: Microstratigraphic preservation of ancient faunal and hominin DNA in Pleistocene cave sediments
Source: Proc Natl Acad Sci U S A. 2021 Dec 27;119(1):e2113666118. doi: 10.1073/pnas.2113666118 (PMC8740756; doi:10.1073/pnas.2113666118)
Supplement: Supplementary File [file pnas.2113666118.sapp.pdf]

Supplementary Information for

Microstratigraphic preservation of ancient faunal and hominin DNA in Pleistocene cave sediments

Diyendo Massilani[1], Mike W. Morley[2], Susan M. Mentzer[3], Vera Aldeias[4], Benjamin Vernot[1], Christopher Miller[3,5], Mareike Stahlschmidt[1], Maxim B. Kozlikin[6], Michael V. Shunkov[6], Anatoly P. Derevianko[6], Nicholas J. Conard[3], Sarah Wurz[5,7], Christopher S. Henshilwood[5,7], Javi Vasquez[8], Elena Essel[1], Sarah Nagel[1], Julia Richter[1], Birgit Nickel[1], Richard G. Roberts[9,10], Svante Pääbo[1], Viviane Slon[1,11,12], Paul Goldberg[3,9], Matthias Meyer[1]

1. Max Planck Institute for Evolutionary Anthropology, D-04103 Leipzig, Germany
2. Archaeology, College of Humanities and Social Sciences, Flinders University, Adelaide, South Australia 5042, Australia
3. Senckenberg Centre for Human Evolution and Paleoenvironment (HEP-Tübingen) and Institute for Archaeological Sciences, Eberhard Karls Universität Tübingen, 72070 Tübingen, Germany
4. Interdisciplinary Center for Archaeology and Evolution of Human Behaviour (ICArEHB), University of Algarve, 8005-139 Faro, Portugal
5. SFF Centre for Early Sapiens Behaviour (SapienCE), University of Bergen, Norway
6. Institute of Archaeology and Ethnography, Russian Academy of Sciences, Siberian Branch, Novosibirsk 630090, Russia
7. School of Geography, Archaeology and Environmental Studies and Evolutionary Studies Institute, University of the Witwatersrand, Johannesburg, South Africa
8. Environmental Management of Military Lands (CEMML), Colorado State University, Fort Collins, CO, 80524, USA
9. Centre for Archaeological Science, School of Earth, Atmospheric and Life Sciences, University of Wollongong, Wollongong, New South Wales 2522, Australia
10. Australian Research Council (ARC) Centre of Excellence for Australian Biodiversity and Heritage, University of Wollongong, Wollongong, New South Wales 2522, Australia
11. Department of Anatomy and Anthropology and Department of Human Molecular Genetics and Biochemistry, Sackler Faculty of Medicine, Tel Aviv University, Tel Aviv 6997801, Israel
12. The Dan David Center for Human Evolution and Biohistory Research, Sackler Faculty of Medicine, Tel Aviv University, Tel Aviv 6997801, Israel

Corresponding authors: Svante Pääbo, Diyendo Massilani, Matthias Meyer, Mike W. Morley
email: paabo@eva.mpg.de, diyendo_massilani@eva.mpg.de, mmeyer@eva.mpg.de, mike.morley@flinders.edu.au

This PDF file includes:

Supplementary text
Figures S1 to S26
Tables S1 to S7
Legends for Datasets S1 to S4
SI References

Other supplementary materials for this manuscript include the following:

Datasets S1 to S4

Supplementary Information Text

Mini-block samples

For the impregnation of sediment into mini-blocks we used loose samples from six Palaeolithic sites selected based on their known preservation of ancient mammalian DNA from previously published records (1, 2) (Dataset S1):

From Slon et al. (2017)(1):

- Sediment sample 19, Chagyrskaya Cave (Russia), sublayer 6c/1
- Sediment sample 67, Denisova Cave (Russia), Main Chamber, layer 21
- Sediment sample 82, Trou Al'Wesse (Belgium), stratum 17b
- Sediment sample 84, Vindija Cave (Croatia), layer G3
- Sediment sample 85, El Sidrón (Spain), stratum III

From Vernot et al. (2021)(2):

- Sediment sample GE-I-A1, Galería de las Estatuas (Spain), Pit I layer 2
- Sediment sample GE-I-A2, Galería de las Estatuas (Spain), Pit I layer 2

Impregnated block samples

We screened for ancient mammalian mtDNA 47 micromorphology block samples from 13 archeological sites around the world.

Aşıklı Höyük n=1, (Turkey)

<u>Sample location; cultural/technological assignment and/or archaeological age estimate; excavation year</u>

- AH-coprolites: Level 4, Area 4GH, Building 2; Aceramic Neolithic, 10.3-10 kyr BP (3); 2011

<u>Impregnation protocol:</u> The samples were air-dried, then cut in half with a sharp knife. One half of each coprolite was embedded in epoxy resin (Araldite) under vacuum and allowed to cure for 24+ hours.

Blombos Cave n=3, (South Africa)

<u>Sediment sample location; cultural/technological assignment and/or archaeological age estimate; excavation year</u>

- BBC 13 10: South Sector, Phase M1, layers CA-CB-CC, Stillbay technocomplex, 72.3 ± 3.8 to 75.1 ± 3.2 ka BP(4); 2013
- BBC 13 17: South Sector, Phase M3, layers CN/CO-CP-CPA, 94.9 ± 4.8 to 108.7 ± 11.8 ka BP (4); 2013
- BBC 25a: square D4; Layers DUN-CA-CB, Stillbay technocomplex, 67.8 ± 4.2 to 72.3 ± 3.8 ka BP (4); 2000

<u>Impregnation protocol:</u> Block samples were cut from profiles and encased in plaster-of-paris bandages. The samples were oven dried for 48h at ca. 40 °C. Once dry the samples were placed in containers and indurated under vacuum with a mixture of unpromoted

polyester resin (Viscovoss N50 S – Voss Chemie GmbH) and styrene (Merck Eurolab GmbH) in a ratio of 7 parts resin to ca. 3 parts styrene, with 5 ml of methyl-ethyl-ketone peroxide added to each liter of resin/styrene mixture. Once the mixture had gelled, they were heated again at 40 °C for 24h to completely harden. The blocks were then sliced with a rock saw using water as a lubricant. For sample BBC 25a a similar protocol was followed; however, the samples were both dried and heated at 60°C instead of 40°C.

Bizmoune n=5, (Morocco)

<u>Sample location (sediment); cultural/technological assignment and/or archaeological age estimate; excavation year</u>

- BIZ-14-01: Sector 2, north profile, reworked sediment from Layer 4 mixed with Later Stone Age materials; broadly late Pleistocene to Holocene; 2014
- BIZ-14-07: Sector 1, south profile, Layer 4c/4b transition; minimum age of 102,980 +/- 9,620 (5); 2014
- BIZ-14-08: Sector 1, east profile, Layer 3 but containing reworked Layer 4 material; mixed ages with a minimum age of 61,820 +- 14,990 (5); 2014
- BIZ-14-11: Sector 4, west profile, Layer 3; minimum age of 61,820 +- 14,990 (5); 2014
- BIZ-14-17: Sector 4, north profile, Layer 4c; minimum age of 102,980 +/- 9,620 (5); 2014

<u>Impregnation protocol:</u> Samples were oven-dried for 5-7 days at ~60 °C. Once dry the samples were placed in containers and indurated under vacuum with a mixture of unpromoted polyester resin (Viscovoss N50 S – Voss Chemie GmbH) and styrene (Merck Eurolab GmbH) in a ratio of 7 parts resin to ca. 3 parts styrene, with 5 ml of methyl-ethyl-ketone peroxide added to each liter of resin/styrene mixture. After the resin had gelled to a firm consistency, the samples were placed in an oven overnight at 60° C and then slabbed and trimmed with a rock saw using water as a lubricant.

Denisova Cave n=6, (Russia)

<u>Sample (sediment): location; cultural/technological assignment and/or archaeological age estimate; excavation year</u>

- DCM1B: Main Chamber layers 11.4/11.2; Initial Upper Palaeolithic, 44 ± 5 to 38 ± 3 ka; 2014
- DCM2A: Main Chamber layer 12.2; Middle Palaeolithic, 70 ± 8 to 58 ± 6 ka; 2014
- DCM2B: Main Chamber layers 12.3/12.2; Middle Palaeolithic, 70 ± 8 to 58 ± 6 ka; 2014
- DCM2C: Main Chamber layers 14.1/12.3; Middle Palaeolithic, 70 ± 8 to 58 ± 6 ka; 2014
- DCE5C: East Chamber layers 11.4/11.3; Middle Palaeolithic, 120 ± 11 to 70 ± 8 ka; 2014
- DCE5 left-overs: East Chamber layers 11.4/11.3; Middle Palaeolithic, 120 ± 11 to 70 ± 8 ka; 2014

<u>Impregnation protocol:</u> Extraction from the stratigraphy of ~20cm/10cm/10cm (tall/wide/depth) sediment blocks covered with plaster of Paris bandages. Dehydration at

40° in an oven, impregnation using Dalchem crystic unpromoted polyester resin diluted with styrene (ratio of 7:4) and catalyzed with methyl ethyl ketone peroxide (12.5 ml per litre of resin/styrene mixture) at the Geoarchaeology Laboratory at the Centre for Archaeological Science, University of Wollongong. After curing, the samples were oven dried overnight at 50 °C and trimmed to 50 × 75 mm « wafers » (6).

Geißenklösterle n=2, (Germany)

Sediment sample location; cultural/technological assignment and/or archaeological age estimate; excavation year

- GK 48-327: Profile E, square 48, Layer GH 18b; Middle Paleolithic, 60 ± 4 ka (7); 2001
- GK 460: Profile E, square 48, Layer GH 20; Middle Paleolithic, 82 ± 9 ka to 60 ± 4 ka (7); 2002

Impregnation protocol: Samples were cut as blocks from the profile and wrapped in plaster-of-paris bandages. They were dried in an oven at ~60˚C for a period of days to weeks depending on the size of the blocks. Once dry the samples were placed in containers and partially indurated with a mixture of unpromoted polyester resin (Viscovoss N50 S – Voss Chemie GmbH) and styrene (Merck Eurolab GmbH) in a ratio of 7 parts resin to ca. 3 parts styrene, with 5 ml of methyl-ethyl-ketone peroxide added to each liter of resin/styrene mixture. The samples were impregnated under vacuum for about 30 minutes at <200 mbar. They were then topped up with the resin/styrene/MEKP mixture over a period of several days. Once the mixture had achieved a gel-like consistency (taking anywhere for 5 days to two weeks) they were heated for ca. 24 at ~60˚C. The hardened blocks were then sliced into 1-2cm-thick slabs with a rock saw, using water as a lubricant (8).

Hohle Fels n=3, (Germany)

Sediment sample location; cultural/technological assignment and/or archaeological age estimate; excavation year

- HF 04 1737: Square 55, Layer GH 9, Middle Paleolithic; 2004
- HF 2269: Layer GH 10/11, Middle Paleolithic; 2002
- HF 12B: Layer GH 1k; Magdalenian, 13,240 + 110 BP (9), 12,370 ± 3ß BP (10), 12,490 ± 70 BP (11); 2000

Impregnation protocol: Block samples were either cut directly from the profile or recovered as monoliths during excavation and encased in plaster-of-paris bandages. The blocks were dried in an oven for 6 weeks at 60 °C prior to impregnation. The samples were partially covered with a mixture of unpromoted poyester resin (Viscovoss N50 S, Voss Chemie GmbH), styrene (Merck Eurolab GmbH) and hardener (methyl-ethyl-ketone peroxide) in volume proportions of 7:3:0.025. The samples were kept under vacuum for 30 minutes at < 200 mbar and then removed and completely immersed in the resin mixture. They were then returned to the vacuum where they were kept for 5 days, followed by a period of heating at 60 °C for 24h. The blocks were then cut using a water-cooled rock saw (9).

Kebara n=3, (Israel)

Sediment sample location; cultural/technological assignment and/or archaeological age estimate; excavation year

- KEB 84 6: square K16a; z=-710; Layer X (?); ~ 61.6±3.6 ka (12); 1984
- KEB 86 21: square Q20d; z=-615; Layer VII; 51.9±3.5 ka (12); 1986
- KEB 06 6: square H13; z=-749; Layer X (?); ~ 61.6±3.6 ka (12); 2006

Impregnation protocol: Samples were oven-dried for 5-7 days at ~60 °C and impregnated with unpromoted polyester resin, diluted with styrene in a ratio of 7:3 and catalyzed with methyl-ethyl-ketone peroxide (MEKP). After the resin had gelled to a firm consistency, the samples were placed in an oven overnight at 60° C and then slabbed and trimmed with a rock saw using water as a lubricant.

Klasies River n=5, (South Africa)

Sediment sample location; cultural/technological assignment and/or archaeological age estimate; excavation year

- KRM-13-05A: Cave 1, Witness Baulk, Rubble Brown Sand (RBS) Member, MSA I; MIS 5e/d (13, 14); 2013
- KRM-13-08A: Cave 1, Witness Baulk, base of the LBS, MSA I; MIS 5e/d (13, 14); 2013
- KRM-13-11: Cave 1b, north profile, contact between the Rubble Sand (RS) and the Dark Carbonised (DC) Members, MSA I and MSA II; undated, but broadly MIS 5; 2013
- KRM-13-17: Cave 1a, Upper Member, Howieson's Poort; ~67,000-58,000 kyr (15); 2013
- KRM-13-19: Cave 1a, Rockfall (RF) Member, non-archaeological deposits; 74,000 kyr; 2013

Impregnation protocol: All samples were stabilized in the profiles using gypsum plaster bandages. The blocks were cut open using a drill and oven-dried for 5-7 days at ~50 °C. Samples KRM-13-11, KRM-13-17 and KRM-13-19 were impregnated with unpromoted polyester resin (Viscovoss N50 S, Voss Chemie GmbH), diluted with styrene (Merck Eurolab GmbH) in a ratio of 7:3 and catalyzed with methyl-ethyl-ketone peroxide (MEKP). After the resin had gelled to a firm consistency, the samples were placed in an oven overnight at 60° C and sliced with a rock saw lubricated with water. Samples KRM-13-05a and KRM-13-08a were first embedded along one exposed block face with epoxy resin (Araldite) to achieve a surface impregnation and thin sections that exactly correspond to the exposed stratigraphy. The surfaces were sliced off using a precision saw with oil lubricant, and the remaining oil was removed from the interior portions of the block using an acetone solution. The blocks were later re-impregnated with unpromoted polyester resin, diluted with styrene in a ratio of 7:3 and catalyzed with methyl-ethyl-ketone peroxide (MEKP) and further processed into a second set of thin sections.

Klipdrift Shelter n=2, (South Africa)

Sediment sample location; cultural/technological assignment and/or archaeological age estimate; excavation year

- KDS 14 03: Quadrate R28c, Units PAZ and PBA/PBB, 59.4 + 4.6 ka (16), Howiesons Poort technocomplex; 2014
- KDS 14 05: Quadrate R28c, Units PBC and PBD, 64.6 ± 4.2 to 65.5 ± 4.8 ka (16), Howiesons Poort technocomplex; 2014

Impregnation protocol: Block samples were cut from profiles and encased in plaster-of-paris bandages. The samples were oven dried for 48h at ca. 40 °C. Once dry the samples were placed in containers and indurated under vacuum with a mixture of unpromoted polyester resin (Viscovoss N50 S – Voss Chemie GmbH) and styrene (Merck Eurolab GmbH) in a ratio of 7 parts resin to ca. 3 parts styrene, with 5 ml of methyl-ethyl-ketone peroxide added to each liter of resin/styrene mixture. Once the mixture had gelled, they were heated again at 40 °C for 24h to completely harden. The blocks were then sliced with a rock saw using water as a lubricant.

La Ferrassie n=14, (France)

Sediment sample location; cultural/technological assignment and/or archaeological age estimate; excavation year

- LAF_300: Western Sector squares I4-I5 Layer 4 ; ~41 – 38 ka^{14}C BP (17, 18); 2013
- LAF_302/1: Western Sector square J3 Layers 6/7a; ~40-32 ka ^{14}C BP/~35-32 ka ^{14}C BP (17, 18); 2013
- LAF_302/2: Western Sector square J3 Layers 6/7a; ~40-32 ka ^{14}C BP/~35-32 ka ^{14}C BP (17, 18); 2013
- LAF_305: Western Sector square I3 Layer 4; ~41 – 38 ka^{14}C BP (17, 18); 2013
- LAF_311: Western Sector square I5 Layer 2; ~74 – 62 ka (17, 18); 2013
- LAF_314: Eastern Sector, Layer I1 previous excavations at the site; 2013
- LAF_322/1: Western Sector Layer 4; ~41 – 38 ka^{14}C BP (17, 18) ; 2013
- LAF_322/2: Western Sector Layers 4 and 3; ~41 – 38 ka ^{14}C BP and ~47 – 43 ka ^{14}C BP (17, 18); 2013
- LAF_404: Western Sector Layers 7b and 6; ~35 – 32 ka ^{14}C BP and ~40 – 32 ka ^{14}C BP (17, 18); 2014
- LAF_406/3: Northern Sector Layers D and C; 2014
- LAF_406/4: Northern Sector Layers D and C; 2014
- LAF_407/1: Northern Sector Layers C and B; 2014
- LAF_407/2: Northern Sector Layers C and B; 2014
- LAF_414/R: Northern Sector Layer A; 2014

Impregnation protocol: Samples were extracted using either plaster of Paris bandages from exposed profiles at the site or soft paper. Samples were processed by Thomas Beckmann (Schwülper-Lagesbüttel, Germany) with dehydration and vacuum impregnation with polyester resin. The samples were trimmed to 9 cm x 6 cm sizes.

Pech de l'Azé IV n=1, (France)

Sediment sample location; cultural/technological assignment and/or archaeological age estimate; excavation year

- PDA4 207: square F14; Layers 7 and 8; ~95±4 ka (19, 20); 2003

Impregnation protocol: Sample was oven-dried for 5-7 days at ~60 °C and impregnated with unpromoted polyester resin, diluted with styrene in a ratio of 7:3 and catalyzed with methyl-ethyl-ketone peroxide (MEKP). After the resin had gelled to a firm consistency, the samples were placed in an oven overnight at 60° C and then slabbed and trimmed with a rock saw using water as a lubricant.

Schöningen n=1, (Germany)

Sediment sample location; cultural/technological assignment and/or archaeological age estimate; excavation year

- FS 1-03: Schöningen 13 II-4, layers 4b-4c, "Feuerstelle" 1; late Lower Paleolithic, MIS 9 (21); 2011

Impregnation protocol: Block sample was collected and enclosed in wooden boxes. The sample was dried at 40 C for 1 day and placed in containers and indurated under vacuum with a mixture of unpromoted polyester resin (Viscovoss N50 S – Voss Chemie GmbH) and styrene (Merck Eurolab GmbH) in a ratio of 7 parts resin to ca. 3 parts styrene, with 5 ml of methyl-ethyl-ketone peroxide added to each liter of resin/styrene mixture. After 5-10 days the sample was heated at 50 C until they were hardened. It was sliced with a water-cooled rock saw (22).

Sierra Diablo n=1, (Texas, USA)

Sediment sample location; cultural/technological assignment and/or archaeological age estimate; excavation year

- Sierra Diablo 13-108: base of Stratum A, Trench 2, Extension 2; Late Archaic Period, approximately 1000 to 900 BCE (23); 2013

Impregnation protocol: Sample was oven-dried for 5-7 days at ~50 °C and placed in containers and indurated under vacuum with a mixture of unpromoted polyester resin (Viscovoss N50 S – Voss Chemie GmbH) and styrene (Merck Eurolab GmbH) in a ratio of 7 parts resin to ca. 3 parts styrene, with 5 ml of methyl-ethyl-ketone peroxide added to each liter of resin/styrene mixture. After the resin had gelled to a firm consistency, the sample was placed in an oven overnight at 60° C and then slabbed and trimmed with a rock saw using water as a lubricant.

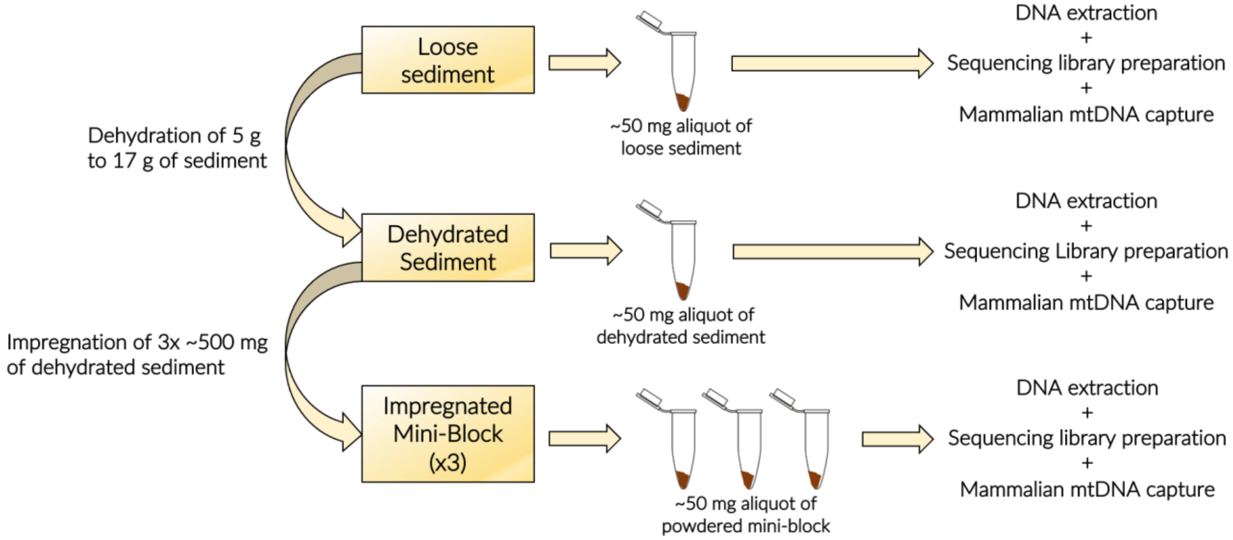

Fig. S1. Overview of the experimental strategy to test whether resin impregnation of sediment interferes with ancient DNA preservation.

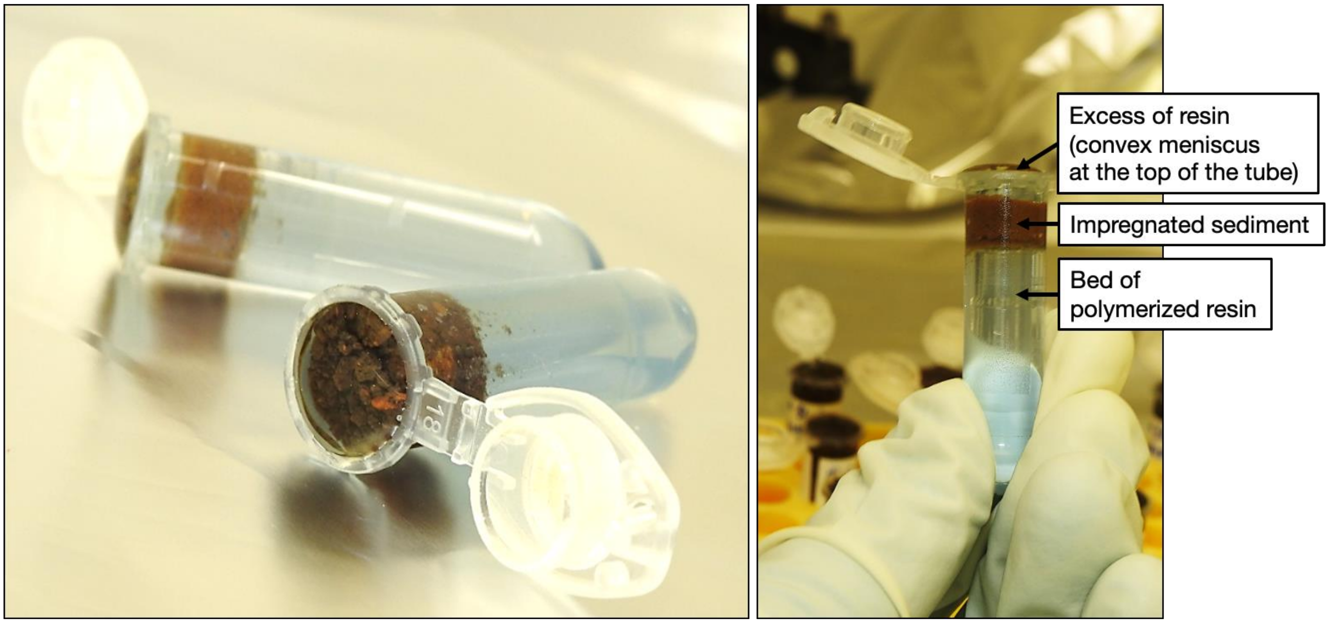

Fig. S2. Sediment impregnation in 2 ml tubes ('mini-blocks'). Picture of mini blocks made by impregnating ~500 mg of dehydrated sediment on top of a hard bed of polyester resin.

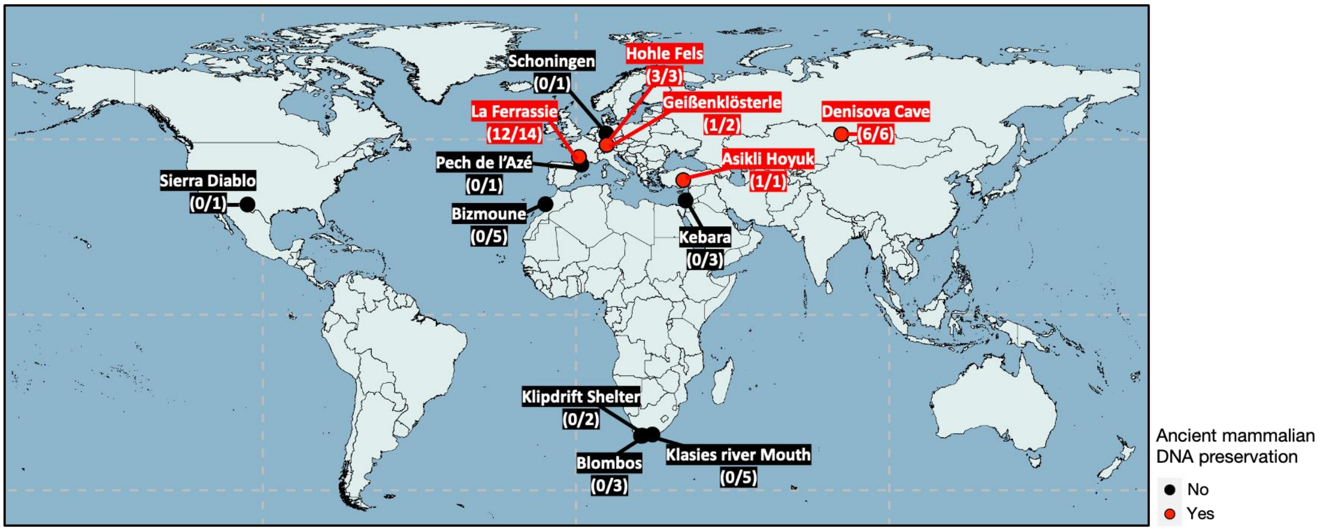

Fig. S3. Location of the 14 sites from which impregnated sediment blocks were screened for ancient mammalian DNA preservation. For each site, the number of positive blocks and the total number of blocks screened are indicated in parentheses. Red color indicates that ancient mammalian DNA was retrieved from at least one micromorphology block from the site.

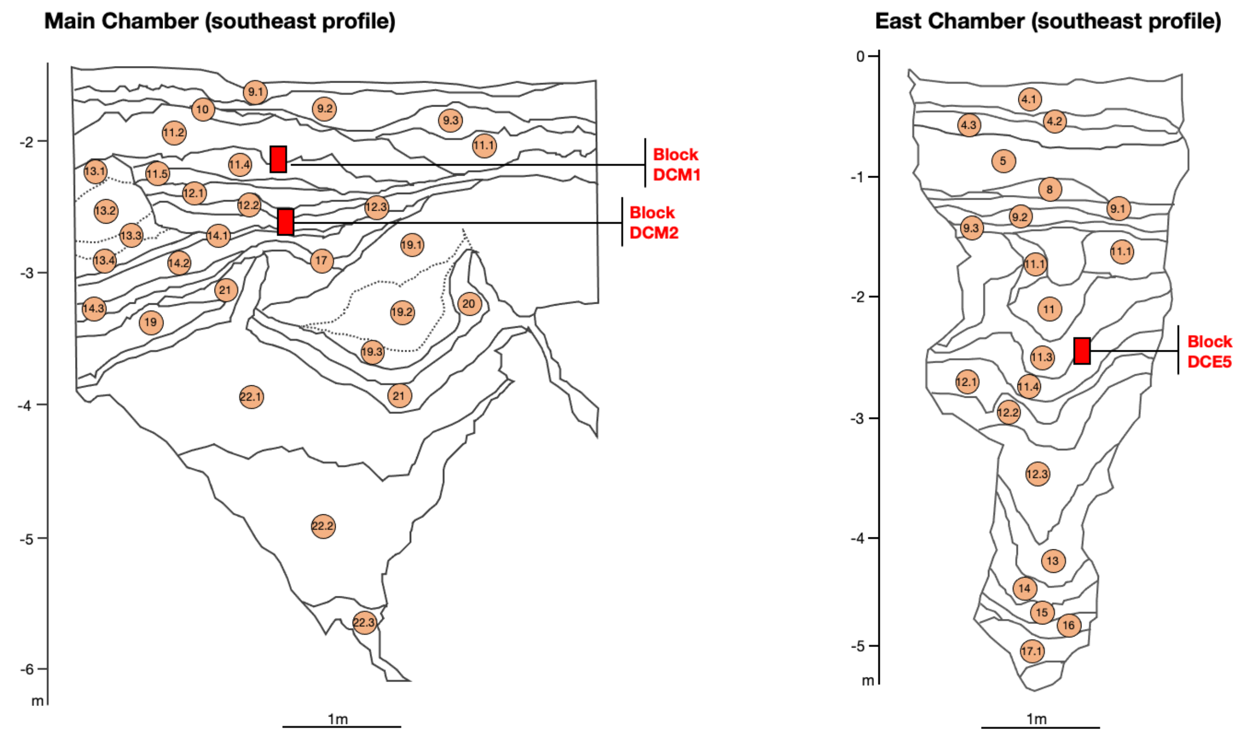

Fig. S4. Locations of the impregnated blocks from Denisova Cave (Russia) used for this study. Stratigraphic sequences showing locations of blocks DCM1 (layers 11.2/11.4) and DCM2 (layers 12.2/12.3/14.1) in Main Chamber and block DCE5 (layers 11.3/11.4) in East Chamber. Numbers in circles denote stratigraphic layers.

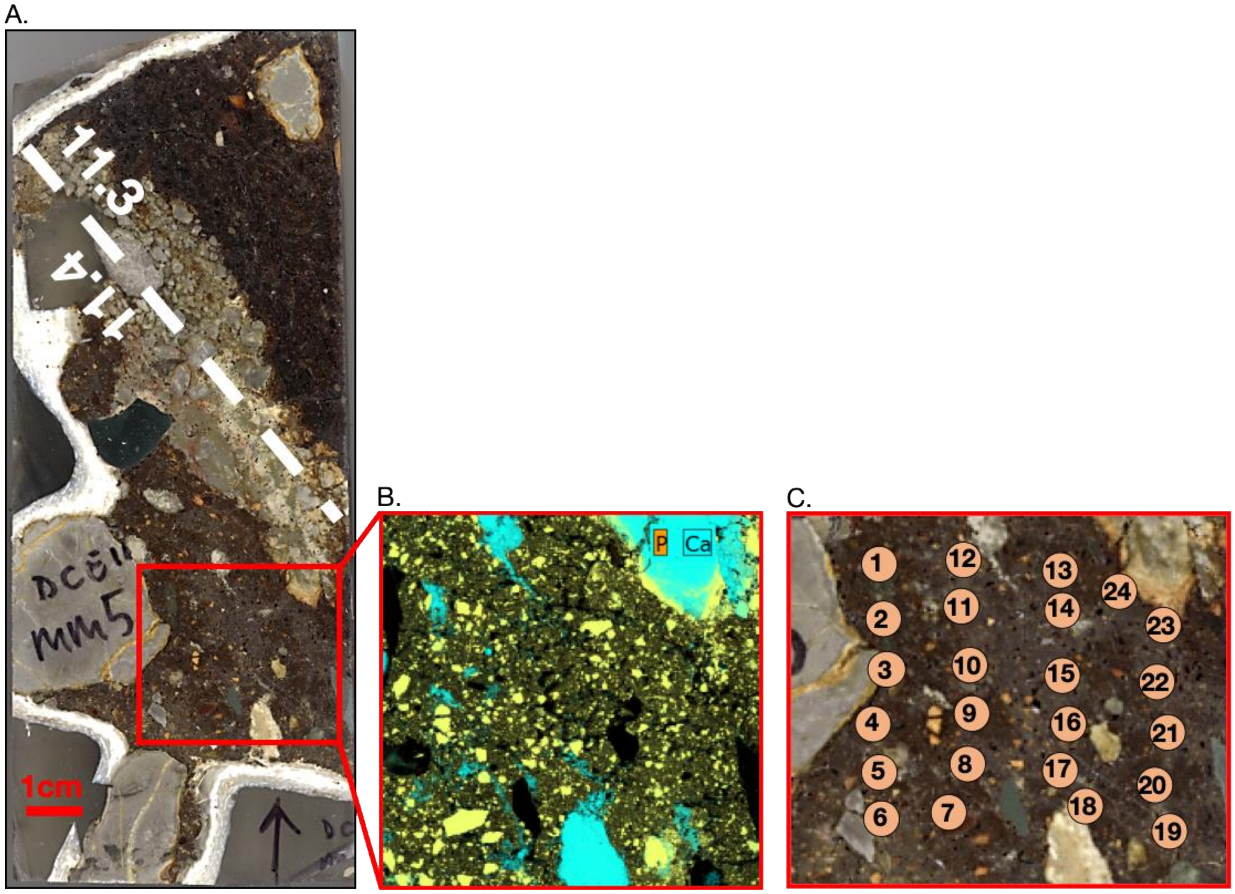

Fig. S5. Grid sampling performed on block DCE5. A) Selected sampling area on block DCE5. **B)** µXRF surface scan for phosphorus (P; orange) and calcium (Ca; aqua) produces a distribution map of calcium phosphate (yellow) that indicates fragments of hydroxyapatite from bone, coprolite and phosphatized limestone. **C)** Location of the 24 spots sampled for DNA analysis in a grid like manner.

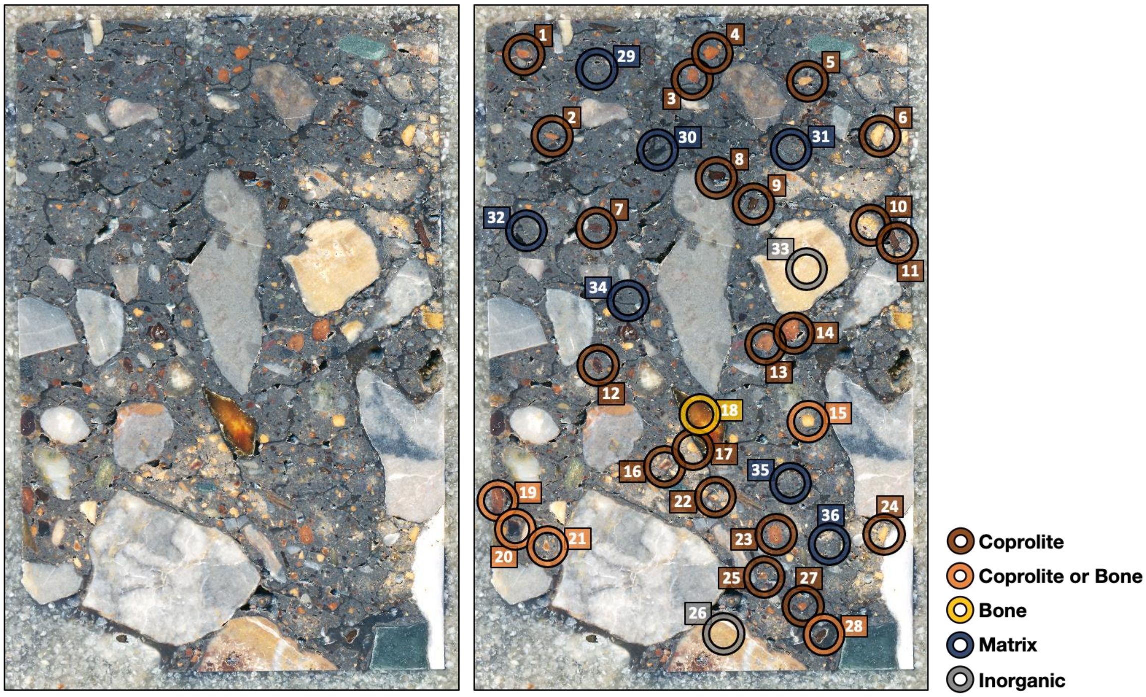

Fig. S6. Cut face of block DCM1B recorded using a flatbed scanner and micro-features sampled for DNA analyses.

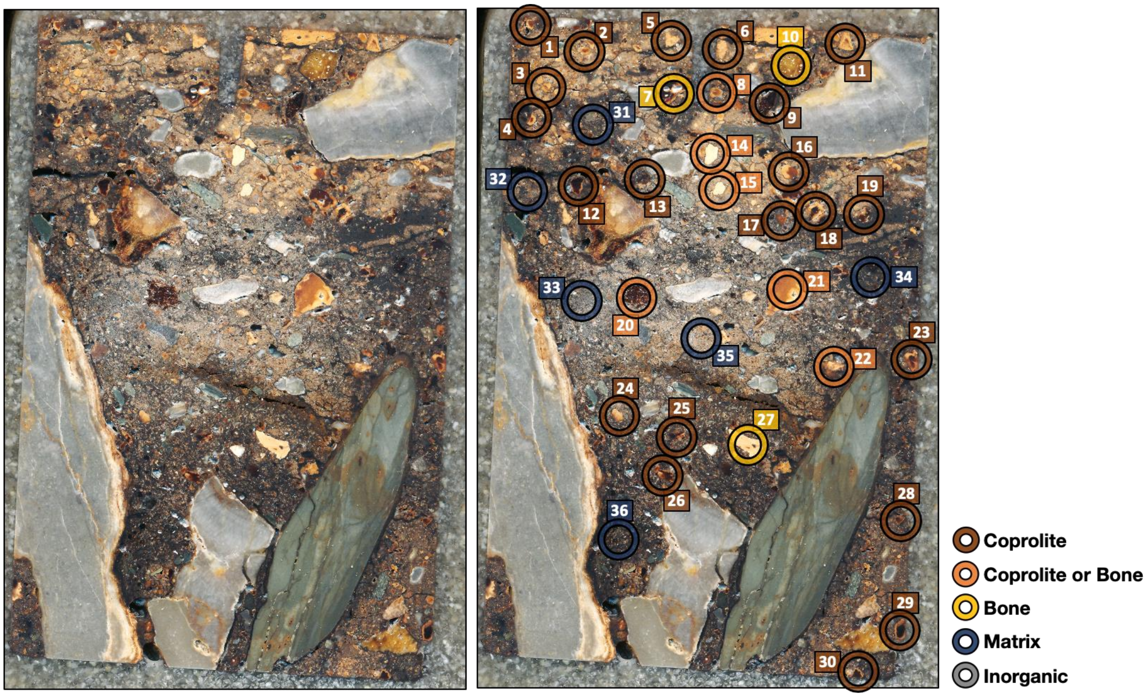

Fig. S7. Cut face of block DCM2A recorded using a flatbed scanner and micro-features sampled for DNA analysis.

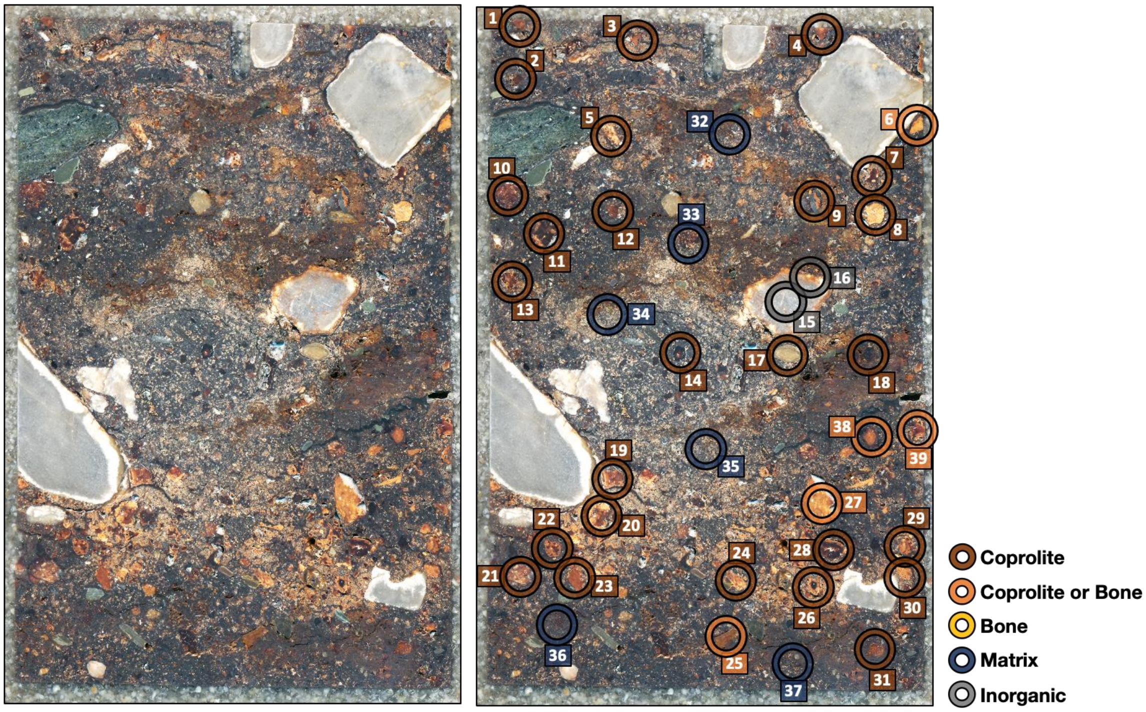

Fig. S8. Cut face of block DCM2B recorded using a flatbed scanner and micro-features sampled for DNA analysis.

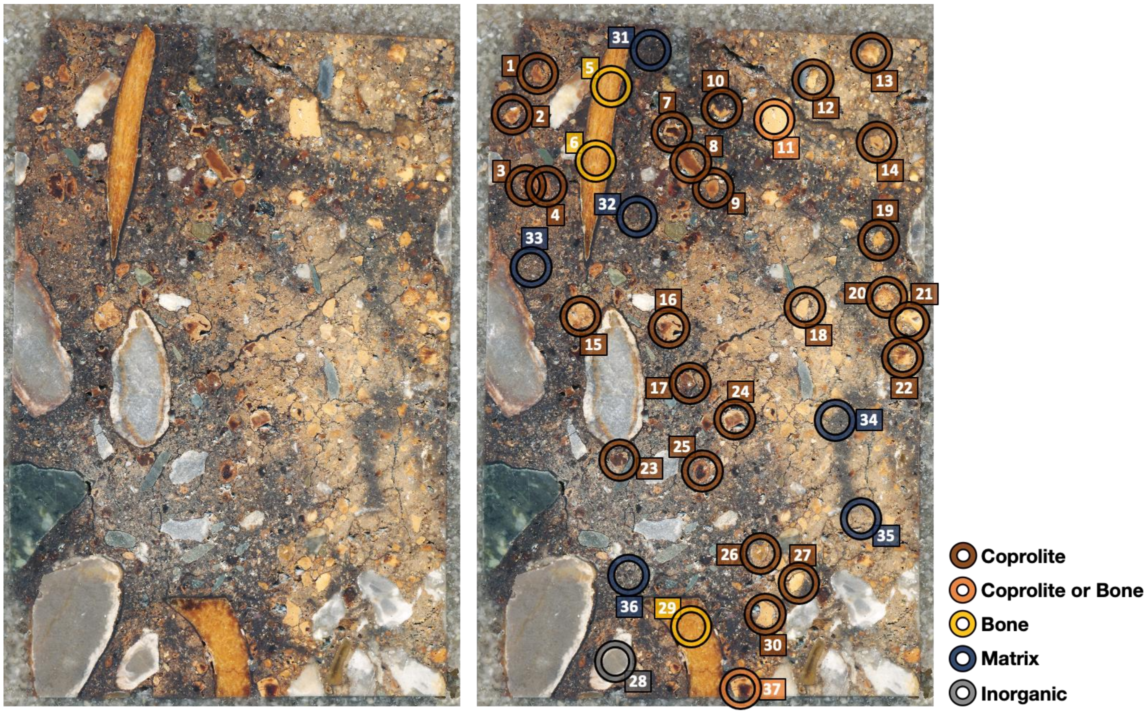

Fig. S9. Cut face of block DCM2C recorded using a flatbed scanner and micro-features sampled for DNA analysis.

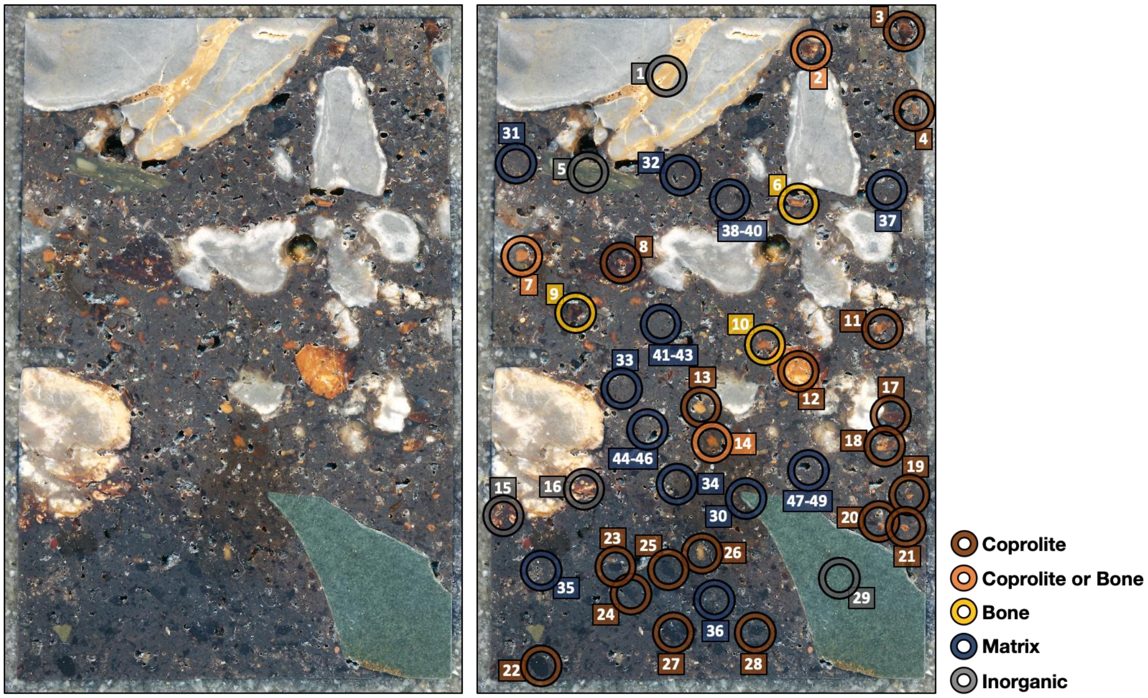

Fig. S10. Cut face of block DCE5C recorded using a flatbed scanner and micro-features sampled for DNA analysis.

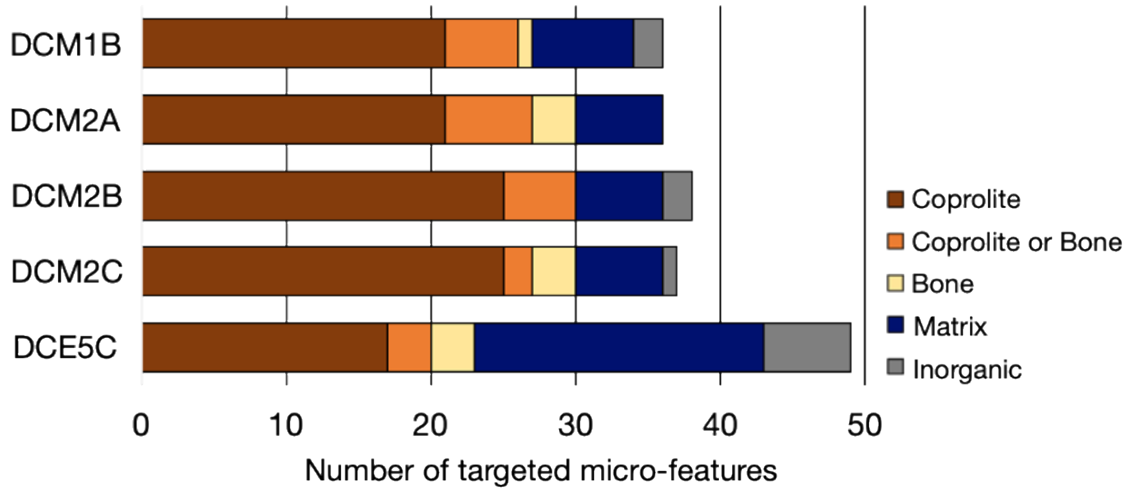

Fig. S11. Type and number of micro-feature and sediment matrix samples targeted in each of the five micromorphological block from Denisova Cave.

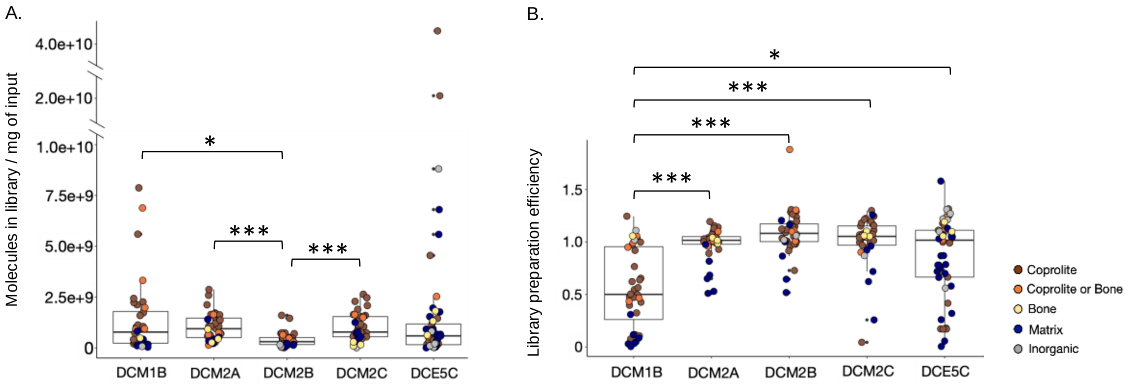

Fig. S12. DNA retrieved from the sediment matrix and micro-features sampled from five micromorphology blocks from Denisova Cave. (A) Boxplots comparing the number of library molecules recovered from the micro-features and sediment matrix samples drilled at the surface of each block, and (B) the efficiency of library preparation. Boxes indicate the mean and interquartile range, whiskers the minimum and maximum values, and outliers are marked with black dots. Differences were tested for significance using an unpaired two-sample Wilcoxon test and considered significant if the p-value was smaller than the significance level alpha = 0.05 after correction was applied from multiple comparisons using the Bonferroni method (p-value: '***' < 0.0001 < '**' < 0.001 < '*'< 0.005).

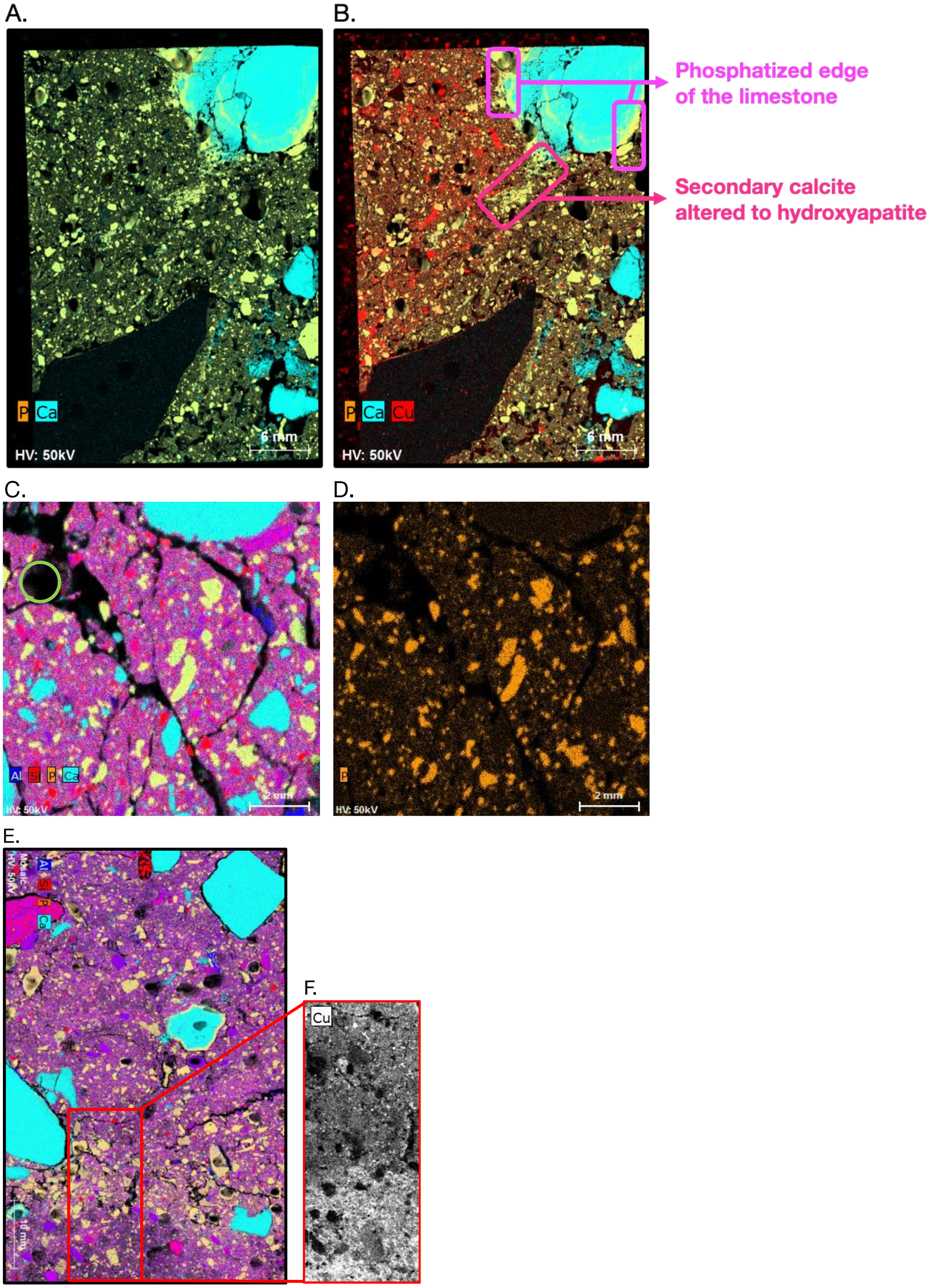

Fig. S13. Micro-XRF surface scan of blocks from Denisova Cave. A) Scan of block DCE5C for phosphorus (P; orange) and calcium (Ca; aqua) which produce a distribution map of calcium

phosphate (yellow) that, in this case, correlates with fragments of hydroxyapatite from bone, coprolite, phosphatized limestone and secondary calcite. Finely divided bone and/or coprolite particles form a part of the sedimentary matrix. **B)** Scan of block DCE5C for copper (Cu; red), phosphorus (P; orange) and calcium (Ca; aqua). The overlap of the three allow a better illustration of the presence of phosphatized limestone edges (magenta frames) and secondary calcite (pink frame). **C)** Scan of a portion of block DCM1B for aluminium (Al; blue) and silicon (Si; red) as illustration of the geogenic components and, phosphorus (P; orange) and calcium (Ca; aqua) showing the distribution of hydroxyapatite from bone, coprolite (calcium-phosphate; yellow). **D)** Scan of a portion of block DCM1B for phosphorus (P) in orange showing that in contrast to limestones in the sediment from layer 11.3 in block DCE5C, the limestones in block DCM1B are not altered to apatite. **F)** Scan of block DCM2B for aluminium (Al; blue), silicon (Si; red), phosphorus (P; orange) and calcium (Ca; aqua). **G)** Distribution map of copper (Cu; white) from the portion of block DCM2B indicated by a red square in **F)**, showing an enrichment of Cu at the base of the block.

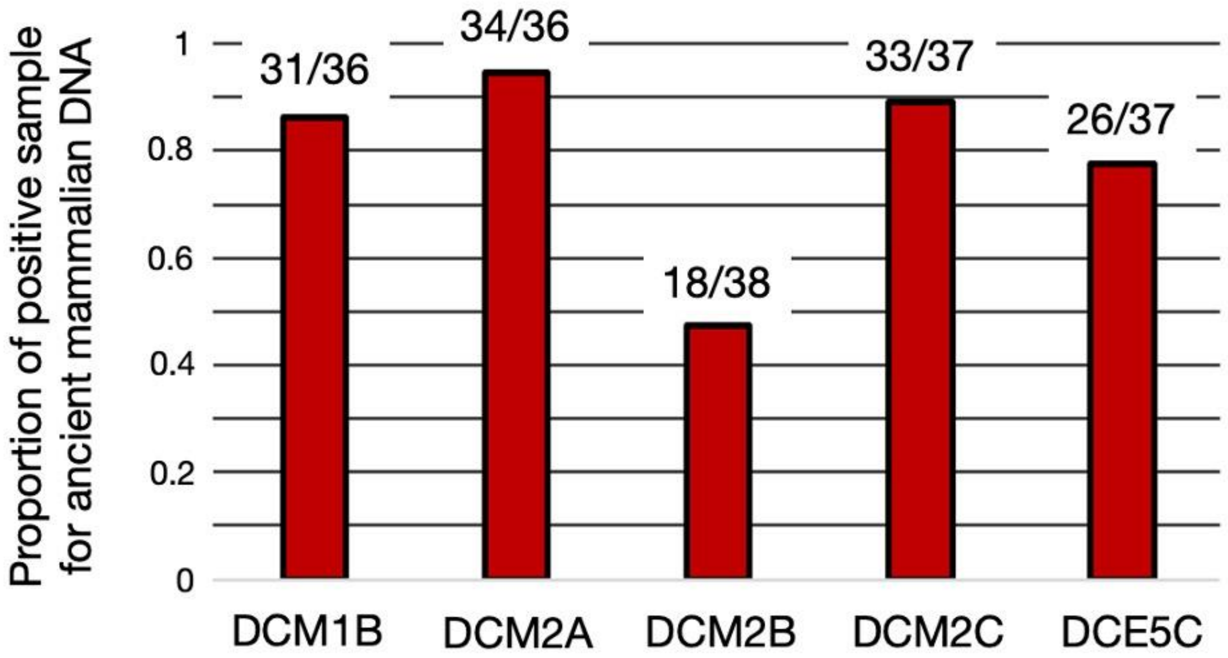

Fig. S14. Proportion of samples positive for ancient mammalian mtDNA in each of the 5 micromorphology blocks from Denisova Cave.

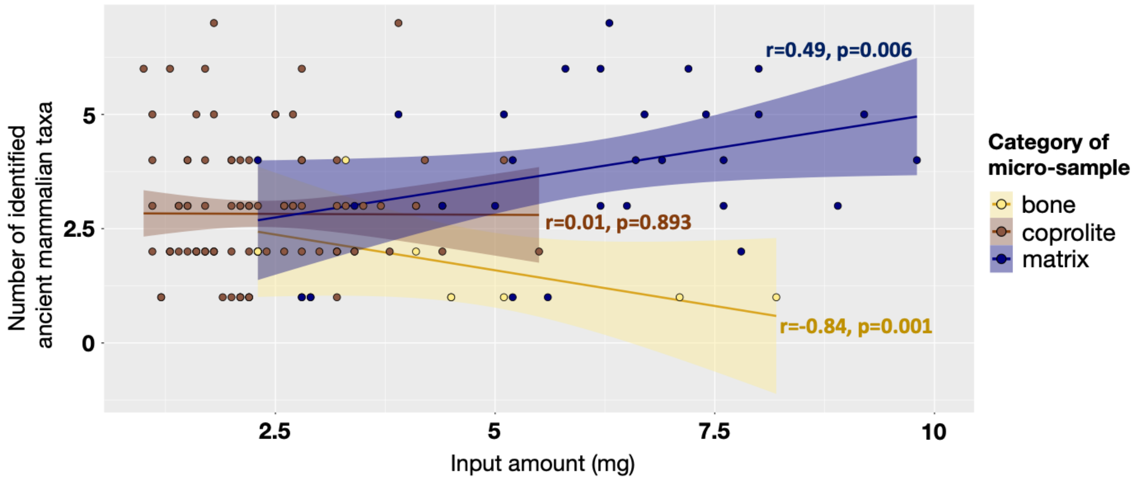

Fig. S15. Correlation between the number of ancient mammalian taxa identified per type of micro-feature and the amount of material sampled. The trend lines represent a simple linear regression for each category of micro-sample (bone (yellow), coprolite (brown), and matrix (blue)) between the number of identified ancient mammalian taxa and the amount of material sampled. The smooth lines represent the 95% confidence interval. For each regression line, the correlation (r) and p-value (p) were estimated using Spearman correlation test. The number of ancient taxa identified in sediment matrix samples tends to increase with the amount of input material. For coprolite samples there is no correlation between both variables; while for bone samples we observed a negative correlation, with a higher number of ancient taxa identified for lower amount of input material. The number of ancient taxa in bone samples approaches a value of 1 for higher amount of input material in agreement with the assumption that the smallest bone samples were contaminated with surrounding sediment during sampling.

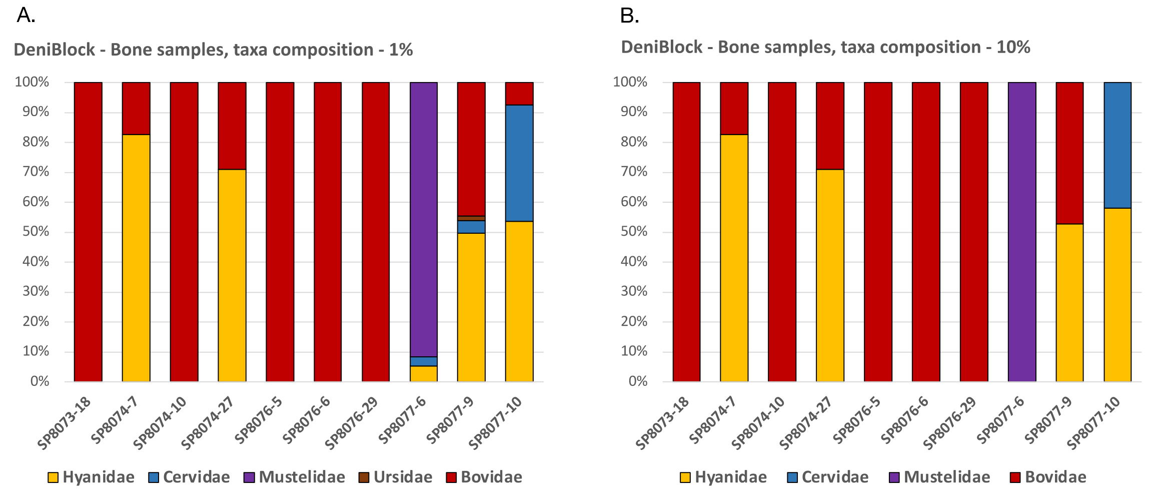

Fig. S16. Ancient mammalian taxa identified in each of the 10 bone micro samples. As the sampling of very small bone micro-features at the surface of the block may have penetrated the underlying sediment, we identified ancient taxa in each bone sample not only **(A)** when represented by at least 1% of the total number of sequences assigned to an ancient taxon but also **(B)** when represented by at least 10% of the total number of sequences assigned to an ancient taxon.

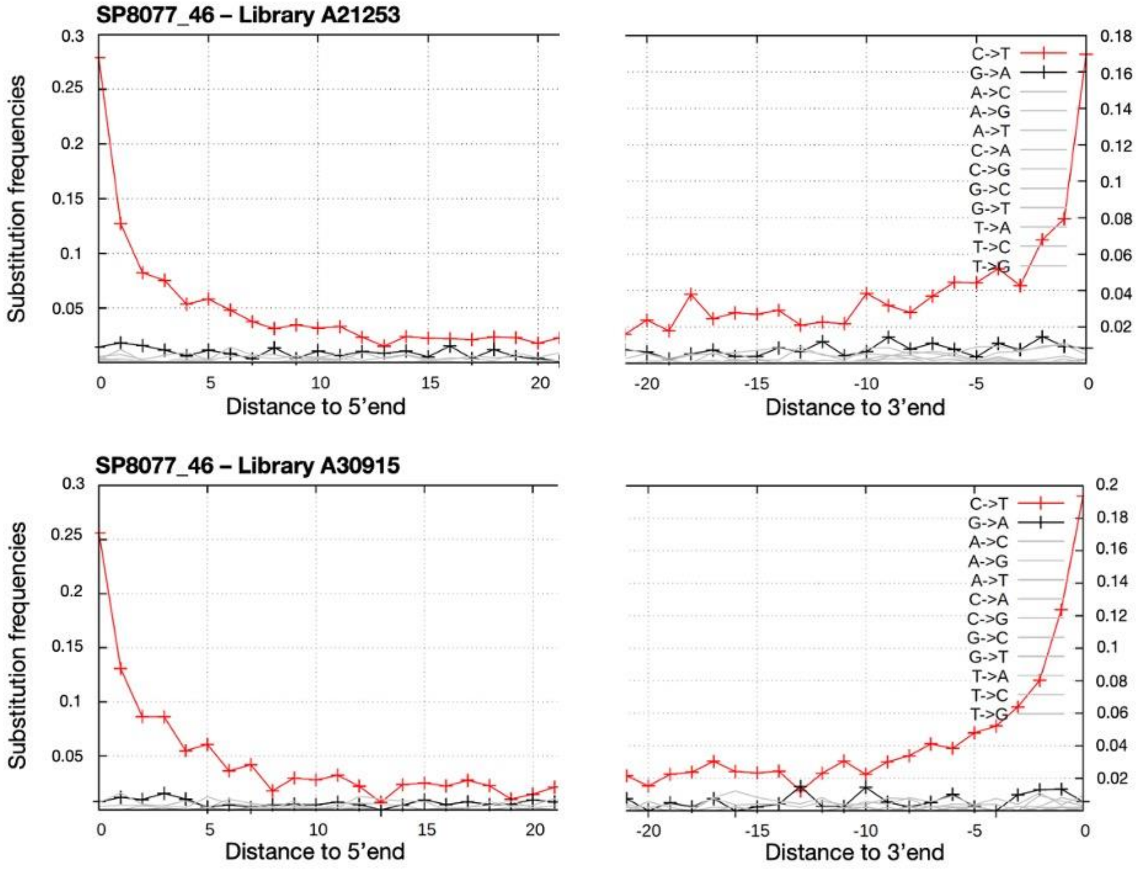

Fig. S17. Substitution frequencies observed in the hominin mtDNA sequences of sample SP8077_46, sediment matrix sample from block DCE5C. Plot of frequencies of base differences between the sequenced fragments and the revised Cambridge reference genome (rCRS) for both libraries of sample SP8077_46. The C to T substitution frequencies are shown in red for the 20 terminal positions.

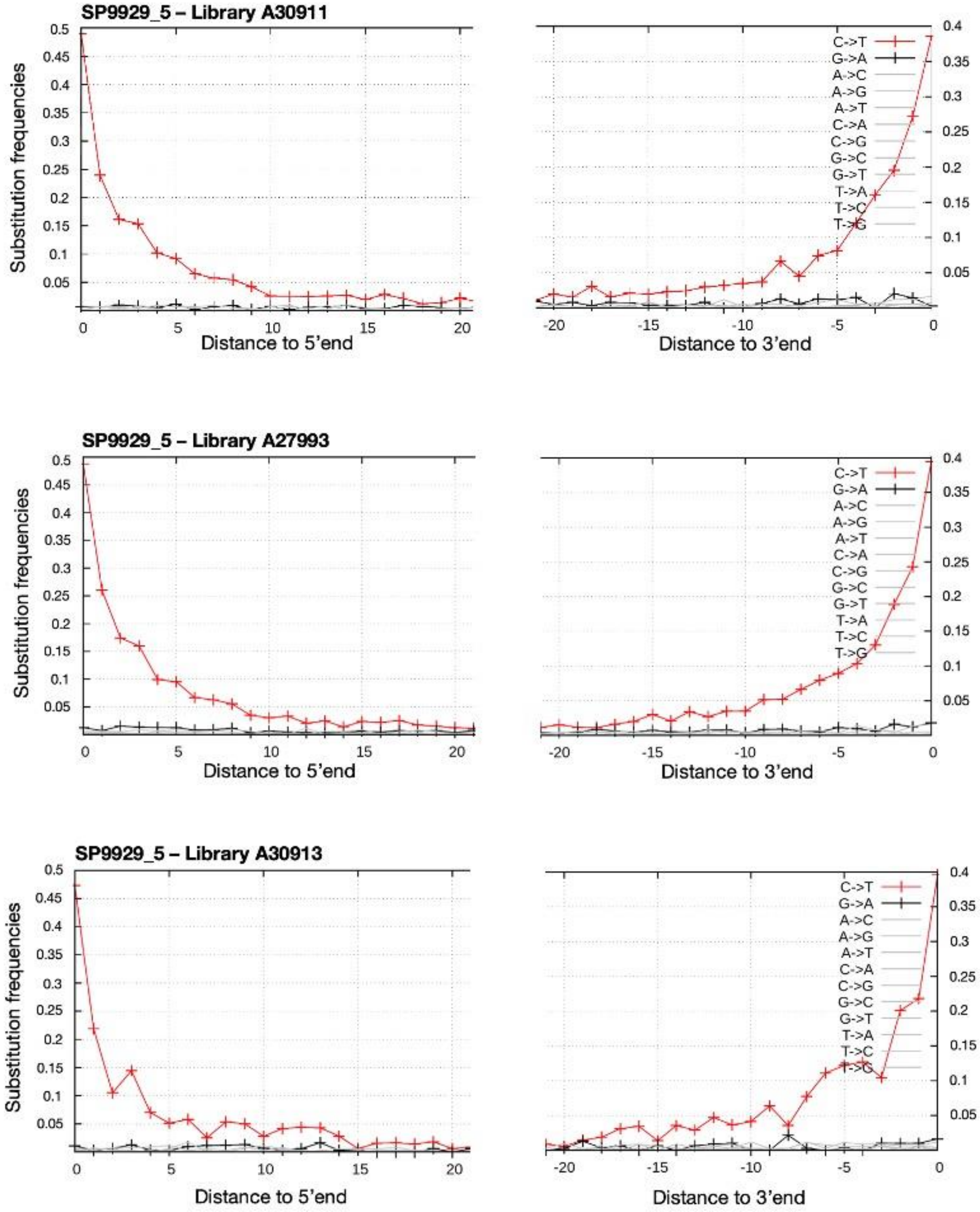

Fig. S18. Substitution frequencies observed in the hominin mtDNA sequences of sample SP9929_5, regular sample from block DCE5D. Plot of frequencies of base differences between the sequenced fragments and the revised Cambridge reference genome (rCRS) for the three libraries of sample SP9929_5. The C to T substitution frequencies are shown in red for the 20 terminal positions.

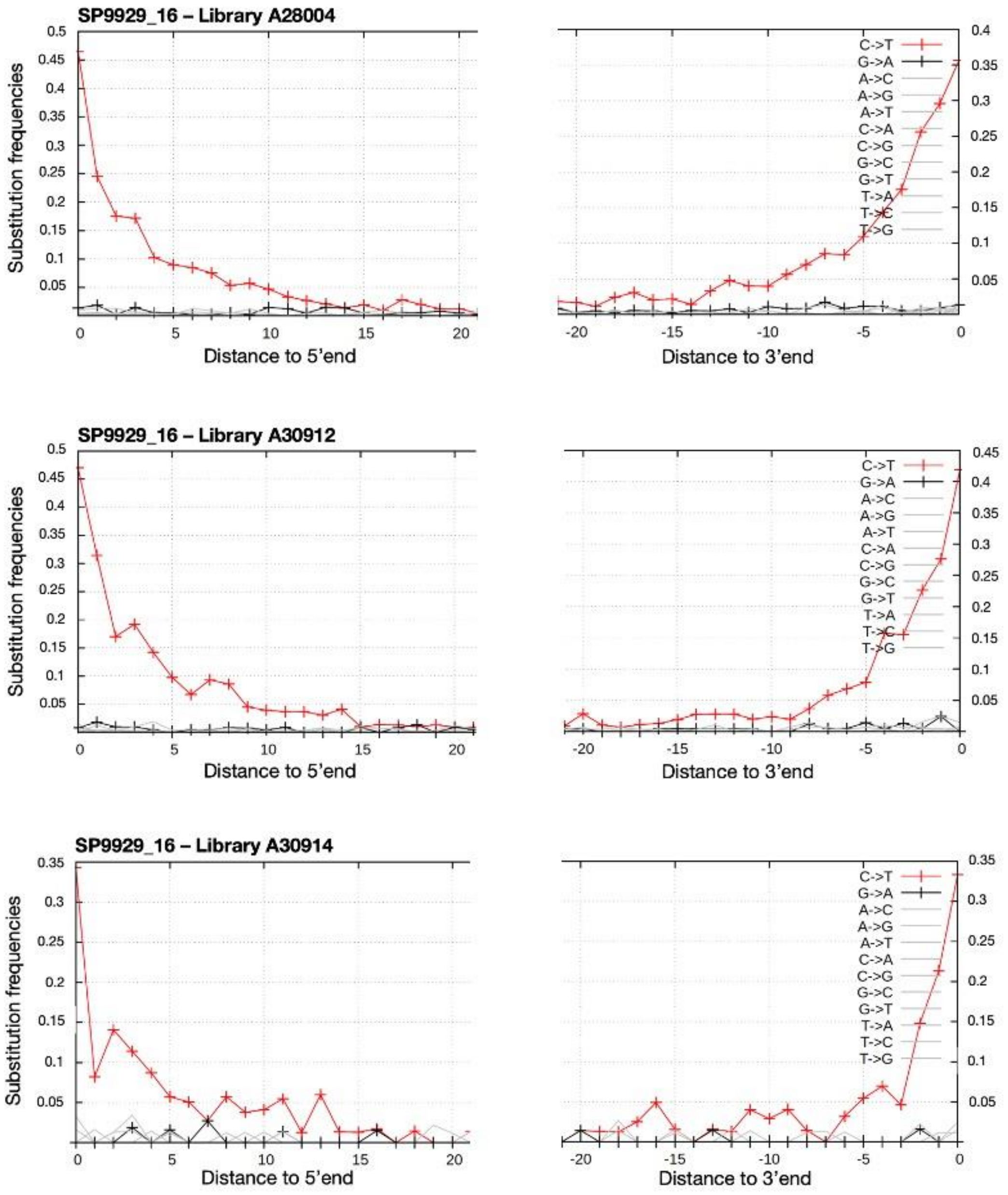

Fig. S19. Substitution frequencies observed in the hominin mtDNA sequences of sample SP9929_16, micro-sample from block DCE5D. Plot of frequencies of base differences between the sequenced fragments and the revised Cambridge reference genome (rCRS) for the three libraries of sample SP9929_16. The C to T substitution frequencies are shown in red for the 20 terminal positions.

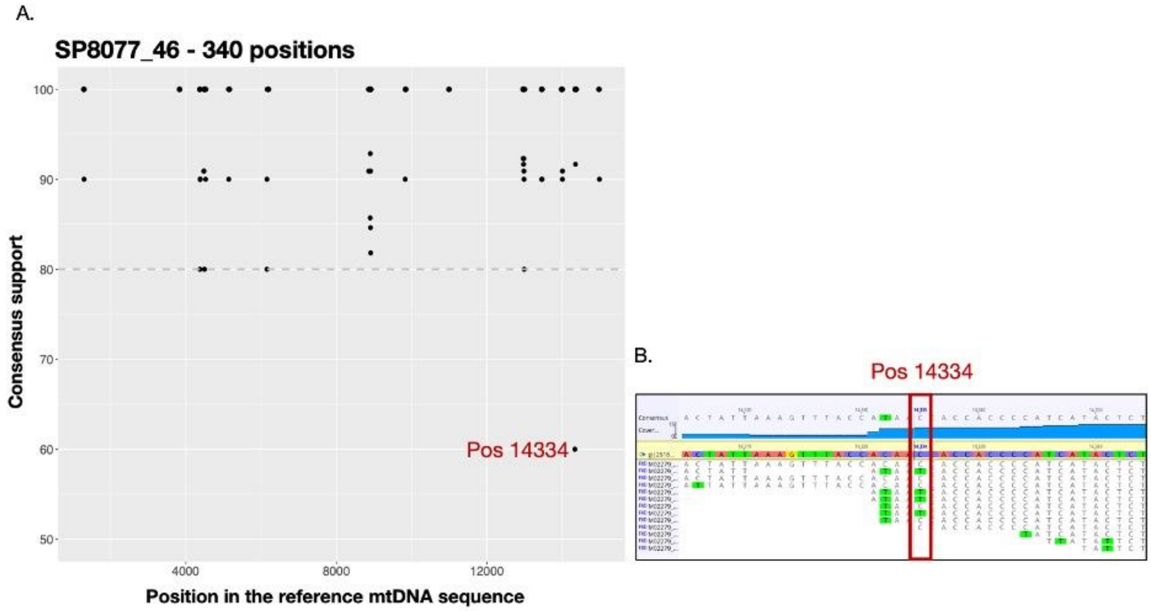

Fig. S20. MtDNA positions covered by at least 10 deaminated sequences from sample SP8077_46, sediment matrix sample from block DCE5C. A. Graph showing the support of the consensus bases at the 340 positions covered by at least 10 deaminated sequences from merged data of the two libraries from sample SP8077_46. Only one position (position 14334 in the rCRS coordinate space) has a consensus base support lower than 80%. **B.** Alignment of sequences from sample SP8077_46 at position 14334, visualized using Geneious Prime 2021.0.3 (https://www.geneious.com). The low support of the consensus base at this position results from C to T mismatches to the reference towards the extremities of the sequences, likely due to cytosine deamination.

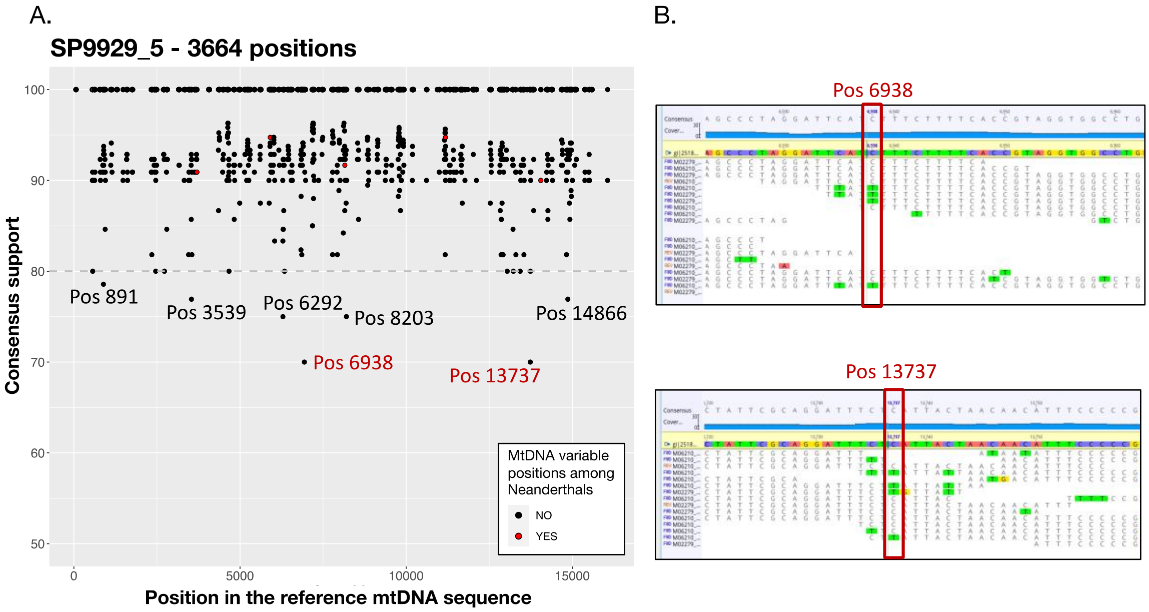

Fig. S21. Human mtDNA positions covered by at least 10 deaminated sequences from sample SP9929_5, regular sample from block DCE5D. A. Graph showing the support of consensus bases at the 3664 positions covered by at least 10 deaminated sequences from merged data of the three libraries from sample SP9929_5. Seven positions have consensus base supports lower than 80%. **B.** Alignment of sequences from sample SP9929_5 at the two positions with the lowest consensus base support (positions 6938 and 13737 in the rCRS coordinate space), visualized using Geneious Prime 2021.0.3 (https://www.geneious.com). The low support of the consensus base at these two positions and the five others with base supports lower than 80% (alignment not shown) result from C to T mismatches to the reference towards the extremities of the sequences, likely due to cytosine deamination.

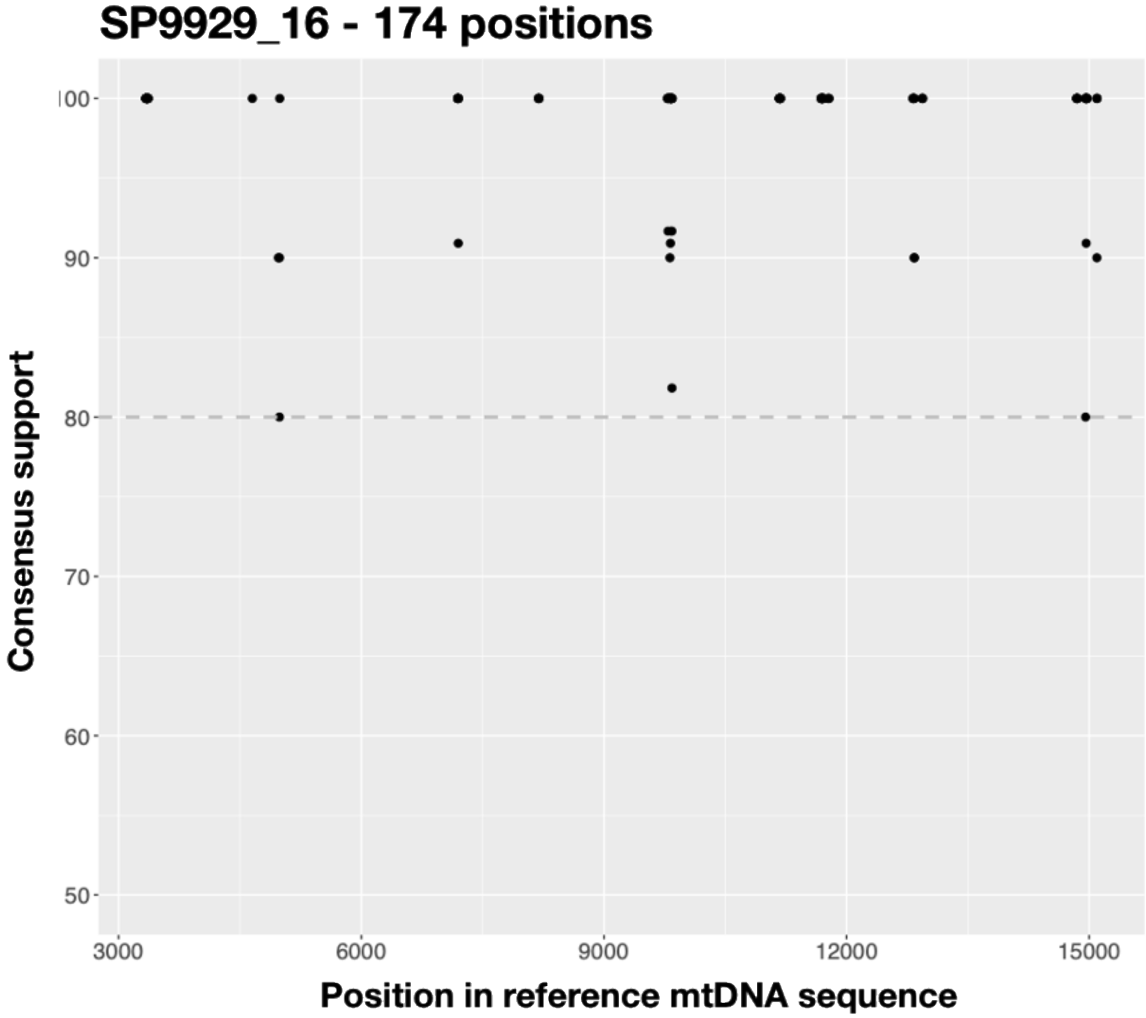

Fig. S22. Human mtDNA positions covered by at least 10 deaminated sequences from sample SP9929_16, micro-sample from block DCE5D. A. Graph showing the support of consensus bases at the 3664 positions covered by at least 10 deaminated sequences from merged data of the three libraries from sample SP9929_16. None of the positions have a consensus base support lower than 80%.

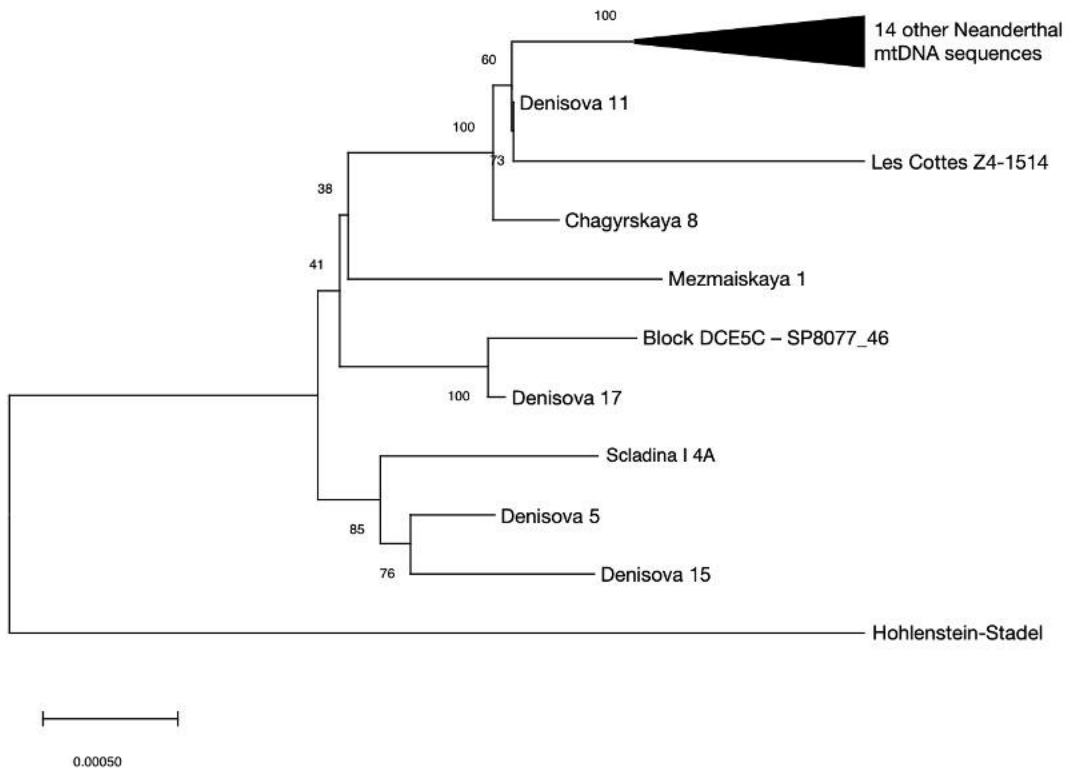

Fig. S23. Neighbor joining phylogenetic tree of 23 previously published Neanderthal mtDNA genome and the mtDNA consensus sequence of the sample from block DCE5C SP8077_46, sediment matrix sample from block DCE5C. The percentage of replicate trees in which the associated taxa clustered together in the bootstrap test (500 replicates) are shown next to the branches with branch length in units of the number of base substitutions per site. All positions containing gaps and missing data were eliminated (complete deletion option). The tree is rooted using the highly divergent mtDNA of the Hohlenstein-Stadel Neanderthal.

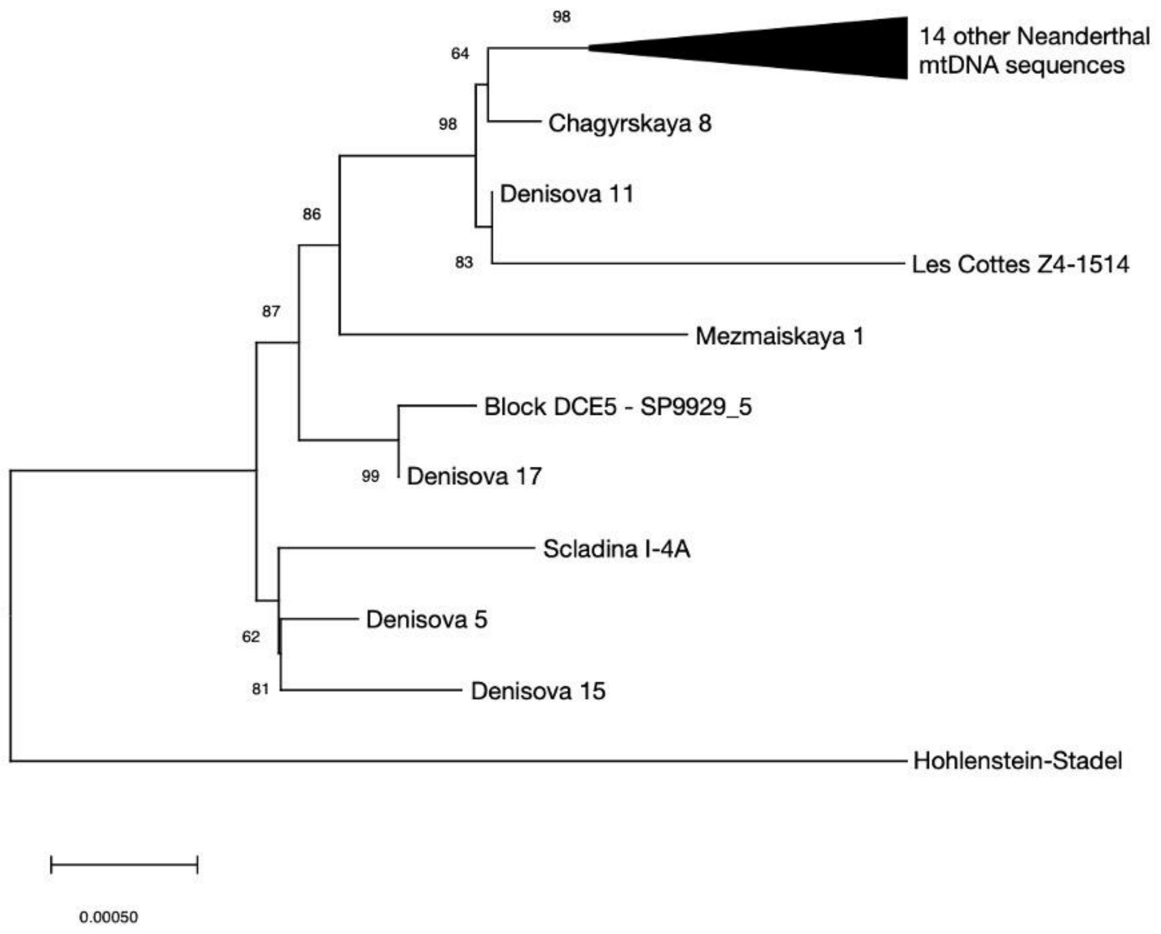

Fig. S24. Neighbor joining phylogenetic tree of 23 previously published Neanderthal mtDNA genome and the mtDNA consensus sequence of the sample from block DCE5 SP9929_5, regular sample from block DCE5D. The percentage of replicate trees in which the associated taxa clustered together in the bootstrap test (500 replicates) are shown next to the branches with branch length in units of the number of base substitutions per site. All positions containing gaps and missing data were eliminated (complete deletion option). The tree is rooted using the highly divergent mtDNA of the Hohlenstein-Stadel Neanderthal.

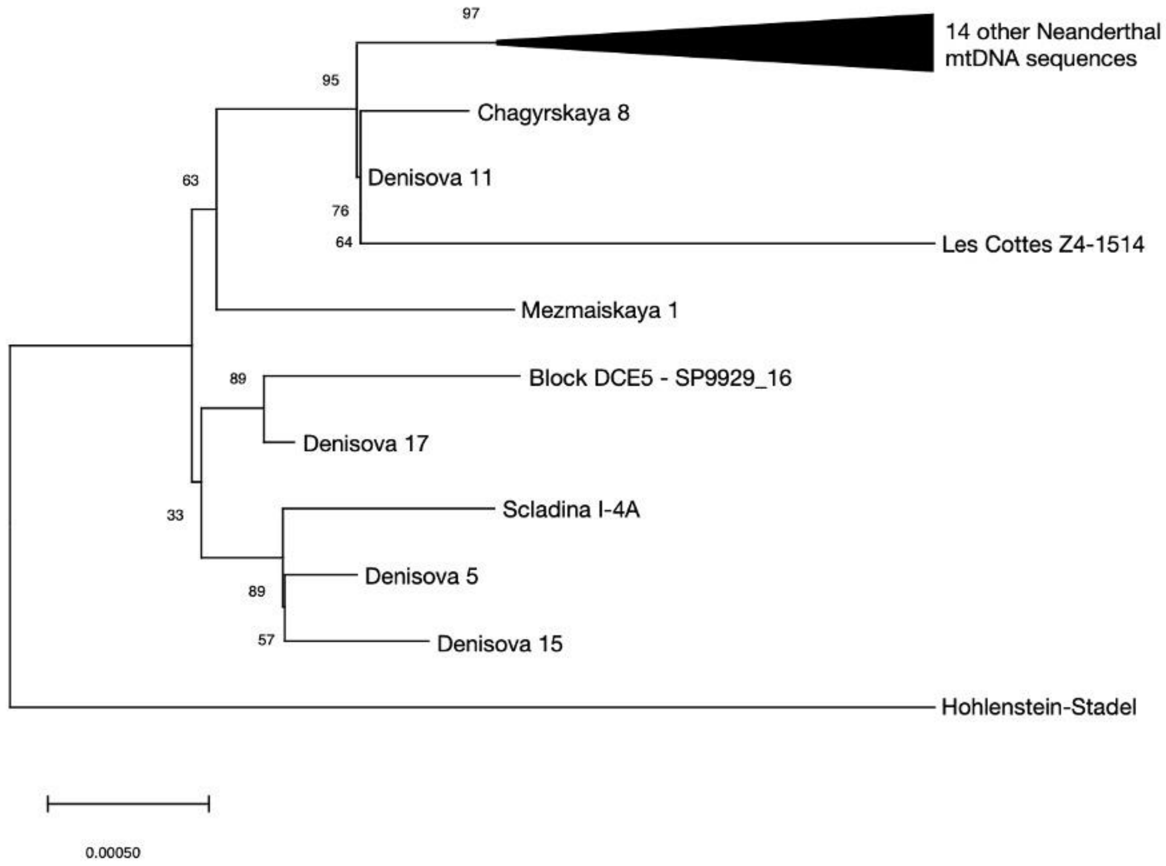

Fig. S25. Neighbor joining phylogenetic tree of 23 previously published Neanderthal mtDNA genome and the mtDNA consensus sequence of the sample from block DCE5 SP9929_16, micro-sample from block DCE5D. The percentage of replicate trees in which the associated taxa clustered together in the bootstrap test (500 replicates) are shown next to the branches with branch length in units of the number of base substitutions per site. All positions containing gaps and missing data were eliminated (complete deletion option). The tree is rooted using the highly divergent mtDNA of the Hohlenstein-Stadel Neanderthal.

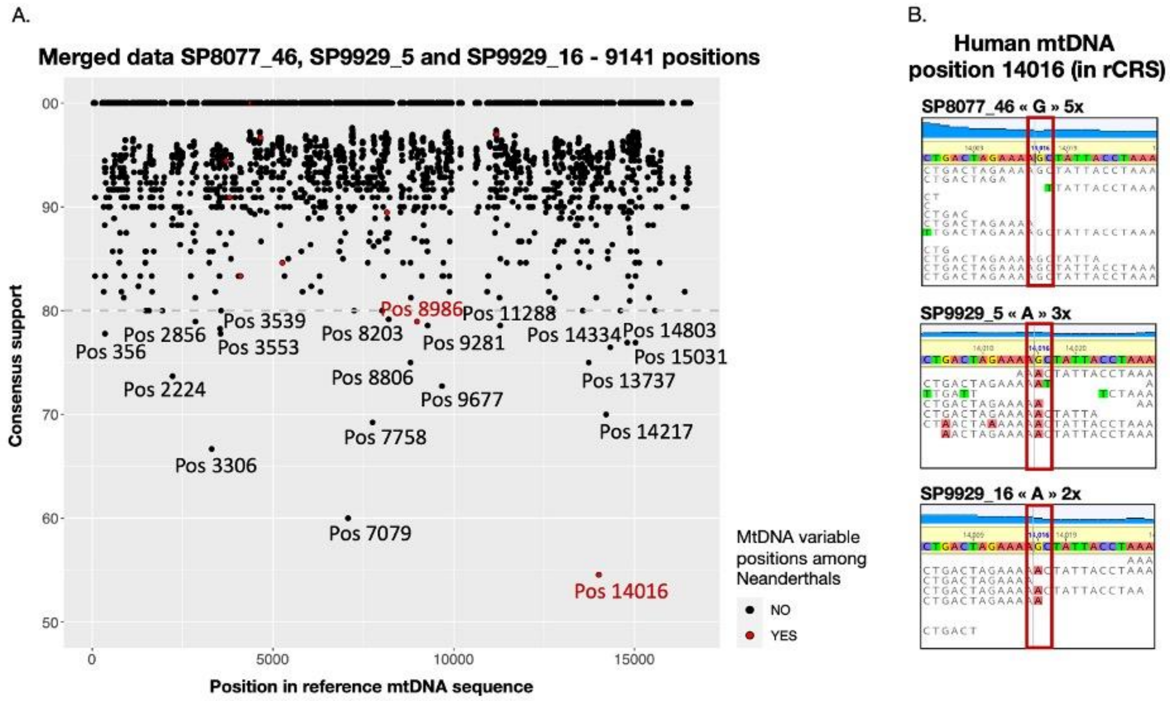

Fig. S26. Human mtDNA positions covered by at least 10 deaminated sequences from merged data of the three samples SP8077_46, SP9929_5 and SP9929_16. A. Graph showing the support of consensus bases at the 9141 positions covered by at least 10 deaminated sequences from merged data of the libraries of the three samples. Twenty positions show a consensus base support lower than 80%, including two positions known to be polymorphic among Neanderthals. With the exception of position 14016, all sequence differences can be explained by deamination. **B.** Alignment of sequences from each of the three samples at position 14016 in rCRS visualized using Geneious Prime 2021.0.3 (https://www.geneious.com).

Table S1. Micromorphology blocks screened for ancient mammalian DNA

	Geographic origin			Number of block samples	Number of blocks positive for ancient mammalian DNA
	Country	Latitude	Longitude		
Bizmoune	Morocco (Africa)	31.51*	-9.76*	5	0
Blombos	South Africa (Africa)	-34.41	21.22	3	0
Klasies River Mouth	South Africa (Africa)	-34.1	24.39	5	0
Klipdrift Shelter	South Africa (Africa)	-34.45	20.73	2	0
Asikli Hoyuk	Turkey (Middle East)	38.34	34.22	1	1
Kebara	Israel (Middle East)	32.57	34.96	3	0
Geißenklösterle	Germany (Europe)	48.39	9.77	2	1
Hohle Fels	Germany (Europe)	48.37	9.75	3	3
La Ferrassie	France (Europe)	44.95	0.93	14	12
Pech De L'Azé	France (Europe)	42.77	1.68	1	0
Denisova Cave	Russia (Central Asia)	51.39	84.67	6	6
Schöningen	Germany (Europe)	51.63	9.66	1	0
Sierra Diablo	USA (America)	31.42	-104.9	1	0

*Approximate coordinate

Table S2. Nearest Neighbor value (Rn) of taxon observed in 3 or more regular and micro-samples drilled from block DCE5 from the East Chamber of Denisova Cave.

	Bovidae	Canidae	Cervidae	Elephantidae	Equidae	Hyaenidae	Rhinocerotidae	Ursidae
n	18	11	17	6	6	19	7	13
D(Obs)	2.24	3.18	2.68	3	3.5	2.63	4.86	3.15
**Rn**	**0.51**	**0.56**	**0.59**	**0.39**	**0.46**	**0.61**	**0.69**	**0.61**

n= Number of positive samples for the specified taxon
D(Obs) = mean of the nearest neighbor distance of the specified taxon
Rn = Nearest Neighbor value of the specified taxon

Table S3. Characteristics of the libraries positive for ancient hominin mtDNA

Sample lab ID	Input material for DNA extraction (mg)	DNA extract	Single stranded library lab ID	Number of molecules in library	Library preparation efficiency	Human mtDNA capture library ID	Nbr of sequenced molecules	Mapped Unique seq L>=35	Duplication rate	Unique human seq identified L>=35 MQ>=25	5' C to T freq. [%]	95% CI 5' C to T freq. [%]	3' C to T freq. [%]	95% CI 3' C to T freq. [%]
SP8077_46	30.9	Aliquot 1	A21253	4.18E+09	81.4%	B26715	1061854	14787	17.8	4054	27.9	25.3-30.7	17.0	14.9-19.2
SP8077_46	30.9	Re-extraction	A30915	1.08E+10	88.1%	B36797	126567	5694	2.5	1937	25.6	21.8-29.7	19.4	16.0-23.1
SP9929_5	36.7	Aliquot 1	A27993	9.53E+09	36.9%	B33700	445819	7217	13.9	3457	48.8	45.4-52.3	39.5	36.3-42.8
SP9929_5	36.7	Aliquot 2	A30911	1.31E+10	66.1%	B36793	154003	4924	2.3	2243	49.0	44.8-53.3	38.7	34.7-42.8
SP9929_5	36.7	Re-extraction	A30913	6.91E+09	91.6%	B36795	181728	2973	4.9	1344	47.3	41.7-53.0	39.6	34.4-45.0
SP9929_16	5.6	Aliquot 1	A28004	1.82E+09	64.7%	B33711	541928	2669	58.6	1631	46.5	41.4-51.6	35.6	31.0-40.5
SP9929_16	5.6	Aliquot 2	A30912	2.05E+09	30.9%	B36794	256200	1866	15.7	1088	47.0	41.1-53.0	41.9	36.4-47.6
SP9929_16	5.6	Re-extraction	A30914	1.18E+09	107.4%	B36796	125198	548	16.1	286	34.3	23.2-46.9	33.3	22.2-46.0

ID: Identifier, L = sequence length, MQ = Map Quality, seq = sequence

Table S4. Mitochondrial lineage assignment for the libraries containing ancient hominin mtDNA. The proportion of sequences assigned to a specific group is estimated by dividing the number of sequences sharing the group-specific base at positions allowing to discriminate that group from the others (diagnostic positions) by the total number of sequences overlapping the diagnostic position.

Sample lab ID	Input material for DNA extraction (mg)	DNA extract	Single stranded library lab ID	Human mtDNA capture library ID	Unique human seq identified L>35 MQ>25	Unique deam human seq identified (3 first and last bases)	% Seq. Assigned to modern human		% Seq. Assigned to Neanderthal (including HST)		% Seq. Assigned to Neanderthal (excluding HST)		% Seq. Assigned to Denisovan		% Seq. Assigned to Sima	
							All seq.	Deam seq.	All seq.	Deam seq.	All seq.	Deam seq.	All seq.	Deam seq.	All seq.	Deam seq.
SP8077_46	30.9	Aliquot 1	A21253	B26715	4054	738	35.8% (135/377)	8.3% (3/36)	52.1% (195/374)	86.7% (26/30)	57.0% (118/207)	90.5% (19/21)	0.5% (4/800)	0.0% (0/93)	0.7% (5/682)	0.0% (0/84)
SP8077_46	30.9	Re-extraction	A30915	B36797	1937	352	42.4% (78/184)	0.0% (0/14)	56.2% (100/178)	93.8% (15/16)	55.1% (59/107)	100.0% (7/7)	0.5% (2/416)	4.3% (2/47)	0.6% (2/338)	2.9% (1/34)
SP9929_5	36.7	Aliquot 1	A27993	B33700	3457	1156	15.3% (29/190)	0.0% (0/26)	77.2% (146/189)	96.4% (27/28)	76.7% (66/86)	85.7% (12/14)	1.1% (7/657)	2.1% (3/142)	0.4% (2/493)	0.9% (1/109)
SP9929_5	36.7	Aliquot 2	A30911	B36793	2243	742	16.3% (14/86)	28.6% (4/14)	79.1% (91/115)	94.4% (17/18)	81.0% (47/58)	91.7% (11/12)	0.2% (1/420)	0.0% (0/82)	0.7% (2/289)	0.0% (0/62)
SP9929_5	36.7	Re-extraction	A30913	B36795	1344	406	23.3% (17/73)	0.0% (0/13)	53.2% (42/79)	100.0% (8/8)	33.3% (15/45)	83.3% (5/6)	0.0% (0/248)	0.0% (0/51)	0.5% (1/192)	0.0% (0/37)
SP9929_16	5.6	Aliquot 1	A28004	B33711	1631	537	17.5% (10/57)	0.0% (0/12)	78.5% (51/65)	84.6% (11/13)	73.0% (27/37)	100.0% (4/4)	0.0% (0/306)	0.0% (0/53)	1.5% (3/198)	2.9% (1/35)
SP9929_16	5.6	Aliquot 2	A30912	B36794	1088	412	4.9% (2/41)	0.0% (0/10)	69.8% (30/43)	83.3% (5/6)	76.9% (20/26)	100.0% (5/5)	1.1% (2/185)	0.0% (0/41)	3.6% (5/139)	0.0% (0/28)
SP9929_16	5.6	Re-extraction	A30914	B36796	286	70	60.0% (12/20)	0.0% (0/3)	26.1% (6/23)	100.0% (1/1)	26.7% (4/15)	100.0% (2/2)	0.0% (0/50)	0.0% (0/5)	2.6% (1/39)	0.0% (0/5)

ID: Identifier, L = sequence length, MQ = Map Quality, seq = sequence, deam=deaminated (first and last base only), HST= Hohlenstein-Stadel (most divergent Neanderthal mtDNA)

Table S5. Mitochondrial consensus sequences called from the 3 samples from block DCE5 using deaminated hominin mtDNA fragments.

Sample lab ID	Input material for DNA extraction (mg)	Comments	Unique human seq identified L>35 MQ>25	Unique human deam seq (three first and last base)	mtDNA consensus (deaminated seq only)	
					coverage >= 2 and support >= 66%	average depth coverage
SP8077_46	30.9	Merged 2 libraries	5991	1086	78.70%	3.6x
SP9929_5	36.7	Merged 3 libraries	7044	2271	76.20%	2.4x
SP9929_16	5.6	Merged 3 libraries	3005	1009	53.60%	5.8x

ID: Identifier, L = sequence length, MQ = Map Quality, seq = sequence, deam=deaminated (three first and last base only),

Table S6. Pairwise differences between the 3 Neanderthal mitochondrial consensus sequences from samples of block DCE5 and 23 previously published Neanderthal mitochondrial genome sequences. Values were inferred using MEGA X, all missing positions were removed for each sequence pair (pairwise deletion option).

	SP8077_46 (10518 positions)	SP9929_5 (11364 positions)	SP9929_16 (6570 positions)
Denisova 17	0.09%	0.04%	0.09%
Denisova 5	0.16%	0.11%	0.14%
Denisova 11	0.18%	0.12%	0.15%
Denisova 15	0.20%	0.15%	0.14%
Chagyrskaya 8	0.20%	0.14%	0.17%
Scladina I-4A	0.21%	0.18%	0.18%
Feldhofer 2	0.23%	0.16%	0.20%
Goyet Q305-4	0.24%	0.16%	0.20%
ElSidron 1253	0.24%	0.18%	0.21%
Mezmaiskaya 2	0.24%	0.18%	0.21%
Mezmaiskaya 1	0.25%	0.18%	0.23%
Vindija 33.17	0.26%	0.19%	0.24%
Vindija 33.19	0.27%	0.20%	0.26%
Vindija 33.16	0.27%	0.20%	0.26%
Goyet Q57-2	0.25%	0.18%	0.23%
Feldhofer 1	0.25%	0.21%	0.23%
Vindija 33.25	0.25%	0.18%	0.23%
Spy 94a	0.29%	0.20%	0.27%
Goyet Q374a-1	0.29%	0.21%	0.27%
Goyet Q305-7	0.29%	0.21%	0.27%
Goyet Q56-1	0.29%	0.21%	0.27%
Les Cottes Z4-1514	0.33%	0.26%	0.33%
Hohlenstein-Stadel	0.52%	0.47%	0.40%

Table S7. Characteristics of the libraries captured for ancient hominin nuclear DNA and population split time of the positive samples using Maximum Likelihood Estimates (MLE) method.

Sample lab ID	Input material for DNA extraction (mg)	DNA extract	Single stranded library lab ID	Nbr of on-target sites	Nbr of sites covered (no kraken filter)	Percent hominin (deam only, no kraken filter)	5' C to T freq. [95% CI] (no kraken filter)	3' C to T freq. [95% CI] (no kraken filter)	Ancient nuclear hominin DNA	Nbr sites covered (Primate kraken filter)	Percent hominin (deam only, Primate kraken filter)	5' C to T freq. [95% CI] (kraken filter)	3' C to T freq. [95% CI] (kraken filter)	Ancient nuclear hominin DNA	MLE Branch*	MLE Branching date estimate*	MLE Branching date estimate [95% CI]*
SP8077_46	30.9	Aliquot 1	A21253	10987	10768	99.3% [51.8%-99.7%]	2.6% [2.0%-3.3%]	1.5% [1.0%-2.0%]	No	9461	100.0% [39.8%-100.0%]	1.9% [1.4%-2.6%]	1.0% [0.6%-1.5%]	No			
SP8077_46	30.9	Re-extraction	A30915	10672	10294	98.4% [63.1%-100.0%]	3.1% [2.4%-3.9%]	1.8% [1.3%-2.4%]	No	8919	100.0% [47.8%-100.0%]	2.2% [1.6%-2.9%]	1.2% [0.8%-1.8%]	No			
SP9929_5	36.7	Aliquot 1	A27993	28919	27950	99.7%. [99.1%-100.0%]	36.7% [35.6%-37.9%]	24.4% [23.3%-25.4%]	Yes	20452	100.0% [98.7%-100.0%]	35.3% [33.9%-36.7%]	23.2% [22.0%-24.4%]	Yes	Altai Neanderthal "Denisova 5"	126.26 ka	124.90-127.93 ka
SP9929_5	36.7	Aliquot 2	A30911	26921	25960	99.4% [98.7%-100.0%]	37.9% [36.6%-39.1%]	27.3% [26.2%-28.4%]	Yes	18136	99.9% [98.6%-100.0%]	35.0% [33.5%-36.5%]	25.5% [24.2%-26.8%]	Yes			
SP9929_5	36.7	Re-extraction	A30913	20548	19773	99.5% [97.3%-99.9%]	34.2% [32.9%-35.6%]	24.0% [22.8%-25.2%]	Yes	14557	100.0% [98.0%-100.0%]	32.2% [30.7%-33.8%]	23.1% [21.8%-24.6%]	Yes			
SP9929_16	5.6	Aliquot 1	A28004	4071	3989	100.0% [93.6%-100.0%]	33.0% [29.8%-36.2%]	25.7% [22.9%-28.6%]	Yes	2765	100.0% [85.8%-100.0%]	27.3% [23.7%-31.1%]	22.8% [19.6%-26.3%]	Yes	Altai Neanderthal "Denisova 5"	122.37 ka	116.89-126.94 ka
SP9929_16	5.6	Aliquot 2	A30912	4098	3971	98.4% [95.1%-100.0%]	40.6% [37.4%-43.9%]	32.1% [29.2%-35.1%]	Yes	2475	100.0% [92.6%-100.0%]	38.3% [34.3%-42.3%]	28.3% [24.8%-32.0%]	Yes			
SP9929_16	5.6	Re-extraction	A30914	2387	2241	98.4% [75.3%-100.0%]	11.6% [8.9%-14.7%]	9.6% [7.2%-12.4%]	No	1797	99.0% [66.4%-100.0%]	7.9% [5.5%-10.9%]	8.1% [5.7%-11.0%]	No			

ID: Identifier, deam=deaminated (first and last base only), MLE= maximum likelihood estimates of divergence dates from a Neanderthal population tree.

*Results obtained from merged data from the same sample

Dataset S1 (separate file). Mini-block experiment, characteristics of sediment samples used for the impregnation into "Mini-blocks". Number of molecules incorporated into DNA sequencing libraries, library preparation efficiencies and ancient mammalian taxonomic composition of loose, dehydrated and impregnated sub-samples.

*sub sample from the same mini-block, NA: Not Applicable, ID: Identifier, L = sequence length, MQ = Map Quality, seq = sequence, ENC=Extraction Negative Control

† Efficiency estimates >100% are expected to occur due to noise in qPCR. Only single measurements were performed per sample and only three controls were available to determine the number of spike-in library molecules that correspond to 100% efficiency.

Dataset S2 (separate file). Characteristics of samples drilled from micromorphology blocks for DNA screening. Number of molecules incorporated into DNA sequencing libraries, library preparation efficiencies and ancient mammalian preservation for each blocks from each sites are reported.

NA: Not Applicable, ID: Identifier, L = sequence length, MQ = Map Quality, seq = sequence, ENC=Extraction Negative Control

Dataset S3 (separate file). Characteristics of macro and micro samples drilled from block DCE5 from the East Chamber of Denisova Cave. Number of molecules incorporated into DNA sequencing libraries, library preparation efficiencies and ancient mammalian taxonomic composition of each sample are reported.

NA: Not Applicable, ID: Identifier, L = sequence length, MQ = Map Quality, seq = sequence, ENC=Extraction Negative Control

Dataset S4 (separate file). Characteristics of micro-features sampled from block DCM1B, DCM2A, DCM2B, DCM2C and DCE5C from Denisova Cave. Number of molecules incorporated into DNA sequencing libraries, library preparation efficiencies and ancient mammalian taxonomic composition of each sample are reported.

NA: Not Applicable, ID: Identifier, L = sequence length, MQ = Map Quality, seq = sequence, ENC=Extraction Negative Control

SI References

1. V. Slon, *et al.*, Neandertal and Denisovan DNA from Pleistocene sediments. *Science* **356**, 605–608 (2017).
2. B. Vernot, *et al.*, Unearthing Neanderthal population history using nuclear and mitochondrial DNA from cave sediments. *Science* **372**, eabf1667 (2021). DOI:10.1038/s41586-021-03675-0
3. J. Quade, *et al*., "Summary of Carbon-14 Dating of the Cultural Levels of Aşıklı Project" in *Early Settl. Aşıklı Höyük- Essays Honor Ufuk Esin, Özbaşaran*, M. Özbaşaran, G. Duru, M.C. Stiner, Eds. (Ege Yayinlari 2018), pp. 43–56.
4. Z. Jacobs, B. G. Jones, H. C. Cawthra, C. S. Henshilwood, R. G. Roberts, The chronological, sedimentary and environmental context for the archaeological deposits at Blombos Cave, South Africa. *Quat. Sci. Rev.* **235**, 105850 (2020).
5. E. M. Sehasseh, *et al.*, Early Middle Stone Age personal ornaments from Bizmoune Cave (Morocco). *Sci. Adv.* ***in press.***
6. M. W. Morley, *et al.*, Hominin and animal activities in the microstratigraphic record from Denisova Cave (Altai Mountains, Russia). *Sci. Rep.* **9**, 13785 (2019).
7. M. Richard, *et al.*, New electron spin resonance (ESR) ages from Geißenklösterle Cave: A chronological study of the Middle and early Upper Paleolithic layers. *J. Hum. Evol.* **133**, 133–145 (2019).

8. P. Goldberg, N. J. Conard, C. E. Miller, “Geißenklösterle Stratigraphy and Micromorphology” in *Geißenklösterle: Chronostratigraphie, Paläoumwelt, Und Subsitenz Im Mittel- Und Jungpaläolithikum Der Schwäbischen Alb*, *Tübinger Monographien zur Urgeschichte*, N.J. Conard, M. Bolus, S.C. Münzel, Eds. (Kerns Verlag 2019), pp. 25–62.
9. P. Goldberg, S. Schiegl, K. Meligne, C. Dayton, N. J. Conard, Micromorphology and Site Formation at Hohle Fels Cave, Swabian Jura, Germany. *E&G Quat. Sci. J.* **53**, 1–25 (2003).
10. A. Immel, *et al.*, Mitochondrial Genomes of Giant Deers Suggest their Late Survival in Central Europe. *Sci. Rep.* **5**, 10853 (2015). https://doi.org/10.1038/srep10853
11. C. Posth, *et al.*, Pleistocene Mitochondrial Genomes Suggest a Single Major Dispersal of Non-Africans and a Late Glacial Population Turnover in Europe. *Curr. Biol.* **26**, 827–833 (2016).
12. P. Goldberg, H. Laville, L. Meignen, O. Bar-Yosef, "Stratigraphy and Geoarchaeological History of Kebara Cave" in *Kebara Cave, Mt Carmel, Israel Volume 2*. O. Bar-Yosef, L. Meignen, Eds. (Peabody Museum 2007), pp. 49–84.
13. E. Loftus, J. Sealy, M. J. Leng, J. A. Lee-Thorp, A late Quaternary record of seasonal sea surface temperatures off southern Africa. *Quat. Sci. Rev.* **171**, 73–84 (2017).
14. J. C. Vogel, "Radiometric Dates for the Middle Stone Age in South Africa" in *Radiometric Dates for the Middle Stone Age South Africa*, J. C. Vogel, Eds. (Firenze University Press 2001), pp. 1000–1008.
15. Z. Jacobs, R. G. Roberts, Single-grain OSL chronologies for the Still Bay and Howieson’s Poort industries and the transition between them: Further analyses and statistical modelling. *J. Hum. Evol.* **107**, 1–13 (2017).
16. C. S. Henshilwood, *et al.*, Klipdrift Shelter, southern Cape, South Africa: preliminary report on the Howiesons Poort layers. *J. Archaeol. Sci.* **45**, 284–303 (2014).
17. A. Balzeau, *et al.*, Pluridisciplinary evidence for burial for the La Ferrassie 8 Neandertal child. *Sci. Rep.* **10**, 21230 (2020).
18. S. Talamo, *et al.*, The new 14C chronology for the Palaeolithic site of La Ferrassie, France: the disappearance of Neanderthals and the arrival of *Homo sapiens* in France. *J. Quat. Sci.* **35**, 961–973 (2020).
19. A. Turq, *et al.*, Les fouilles récentes du Pech de l’Azé IV (Dordogne). *Gall. préhistoire* **53**, 1–58 (2011).
20. P. Goldberg et al., “Stratigraphy, Deposits, and Site Formation” in *The Middle Paleolithic Site of Pech de l'Azé IV,* H. L. Dibble, S. J. P. McPherron, P. Goldberg, D. M. Sandgathe, Eds. (Springer 2018), pp. 21–74.
21. D. Richter, M. Krbetschek, The age of the Lower Paleolithic occupation at Schöningen. *J. Hum. Evol.* **89**, 46–56 (2015).
22. M. C. Stahlschmidt, *et al.*, On the evidence for human use and control of fire at Schöningen. *J. Hum. Evol.* **89**, 181–201 (2015).
23. J. J. Vasquez, "Preliminary archaeological investigations at the Sierra Diablo Cave site: Paleoindian and Archaic occupations in Hudspeth County, Texas" (2010). *ETD Collect. Univ. Texas, El Paso*. AAI1483837.
